# Postharvest preservation of fruits and vegetables by light-emitting diodes

**DOI:** 10.1080/15592324.2026.2684157

**Published:** 2026-06-09

**Authors:** Aradhika Vijeev, Shaik Basha, Spandana S. Nadig, Krishna Kishore Mahato

**Affiliations:** a Department of Biophysics, Manipal School of Life Sciences, Manipal Academy of Higher Education, Manipal, India

**Keywords:** UV-LED, ultraviolet radiation, postharvest biology, antioxidants, fruit ripening

## Abstract

Postharvest losses of fruits and vegetables remain a major challenge because of rapid physiological deterioration, microbial spoilage, and quality degradation during storage and distribution. Light-based technologies, particularly light-emitting diodes (LEDs) and ultraviolet (UV) irradiation, have emerged as promising non-thermal and residue-free approaches for preserving postharvest quality through precise regulation of wavelength, intensity, and exposure duration. However, reported responses remain highly variable across species, cultivars, developmental stages, and treatment conditions, limiting the establishment of predictive and standardized strategies. Previous reviews have focused primarily on either technological applications or specific mechanistic pathways, whereas an integrated understanding across preharvest and postharvest systems remains limited. This review provides a comparative and comprehensive analysis of LED- and UV-mediated regulation across the preharvest–postharvest continuum. Specifically, it synthesizes evidence on the light regulation of fruit growth and development, spectral effects on photosynthesis, metabolism and molecular pathways, enhancement of antioxidants and bioactive compounds, postharvest shelf-life extension, microbial control, senescence regulation, and quality preservation. Additionally, this review critically evaluates the methodological variability, translational limitations, and challenges associated with commercial implementation. Unlike existing reviews, this work integrates wavelength-specific physiological, biochemical, and molecular responses with practical considerations and cross-study comparisons to identify inconsistencies and emerging trends. By emphasizing mechanistic understanding, evidence-based synthesis, and future research priorities, this review provides a framework for developing predictive, crop-specific, and scalable light-based preservation strategies that may contribute to sustainable postharvest management and reduced food losses in horticultural systems.

## Graphical abstract

**Figure:**
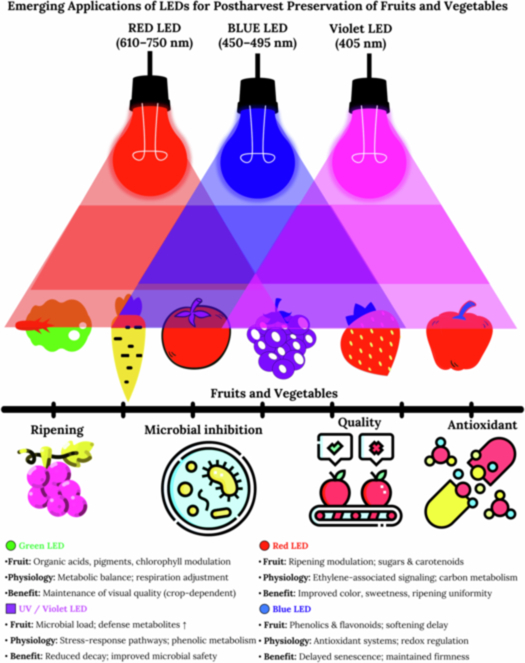

## Introduction

Fruits and vegetables are essential components of the human diet, providing micronutrients, dietary fiber, antioxidants, and bioactive phytochemicals that contribute to the prevention of chronic diseases, including diabetes, cardiovascular disorders, and neurodegenerative disorders.^1-4^ However, their high-water content and continued metabolic activity after harvest make them highly perishable, leading to substantial postharvest losses through microbial spoilage, enzymatic browning, moisture loss, and physiological senescence.^5^ Conventional preservation approaches, such as refrigeration, modified-atmosphere storage, and chemical treatments, can delay deterioration but are often associated with high energy consumption, environmental concerns, and potential compromises in nutritional and sensory quality.^6^ These limitations have driven increasing interest in sustainable, non-chemical preservation strategies.

Among emerging technologies, light-based treatments, particularly light-emitting diodes (LEDs)^7^ ^,^ ^8^ and ultraviolet (UV) irradiation, have gained attention as non-thermal, residue-free approaches that allow precise control over wavelength, intensity, and exposure duration.^9^ ^,^ ^10^ A growing body of research demonstrates that different spectral regions, including blue, red, far-red, and UV wavelengths, can regulate key physiological and biochemical processes such as ripening, senescence, antioxidant metabolism, microbial stability, and energy regulation.^11^ ^,^ ^12^ However, these effects are not uniform across commodities or experimental conditions. While certain crops, such as berries, grapes, and tomatoes, often show enhanced phytochemical accumulation or delayed deterioration under optimized light treatments, as reported in previous studies,^13^ ^,^ ^14^ other commodities exhibit variable or even contradictory responses under similar spectral conditions.^11^ These inconsistencies highlight a central challenge in the field: light-mediated responses are strongly dependent on species, cultivar, developmental stage, and treatment parameters, limiting the generalization of findings.^15^

Recent reviews have addressed different aspects of light-based preservation. For example, Yao et al.^13^ provided a broad overview of multiple light technologies (including LED, UV, fluorescent, and pulsed light) and their applications in fruit and vegetable preservation, while Obajuluwa and colleagues^14^ focused specifically on LED-mediated mechanisms related to fungal decay control and functional quality improvement. Although these studies provide valuable insights, they primarily emphasize either technological applications or specific mechanistic pathways. The systematic integration of wavelength-specific physiological responses, metabolic regulation, and translational feasibility across both preharvest and postharvest stages remains limited.

Importantly, emerging evidence indicates that light effects extend beyond postharvest treatments alone. Preharvest lighting conditions can influence postharvest physiology by modulating metabolic programming, carbon partitioning, stress responses, and nutrient composition, thereby affecting subsequent storage performance.^15^ Recent advances further suggest that the influence of light extends beyond primary metabolism to include the regulation of pigment biosynthesis, volatile profiles, and secondary metabolic pathways, which are often mediated through photoreceptor signaling networks and downstream transcriptional regulation.^16^ In addition to these conceptual gaps, methodological variability presents a major limitation. Differences in spectral composition, irradiance, exposure duration, environmental conditions, and experimental design hinder cross-study comparisons and reduce the reproducibility of reported outcomes. Furthermore, most studies are conducted under controlled laboratory conditions, with limited evaluation under commercial storage and supply chain environments. Critical factors such as scalability, energy efficiency, cost-effectiveness, and integration with existing postharvest systems are rarely assessed systematically. These limitations collectively prevent the development of predictive and standardized light-based preservation strategies.

**Figure 1.**
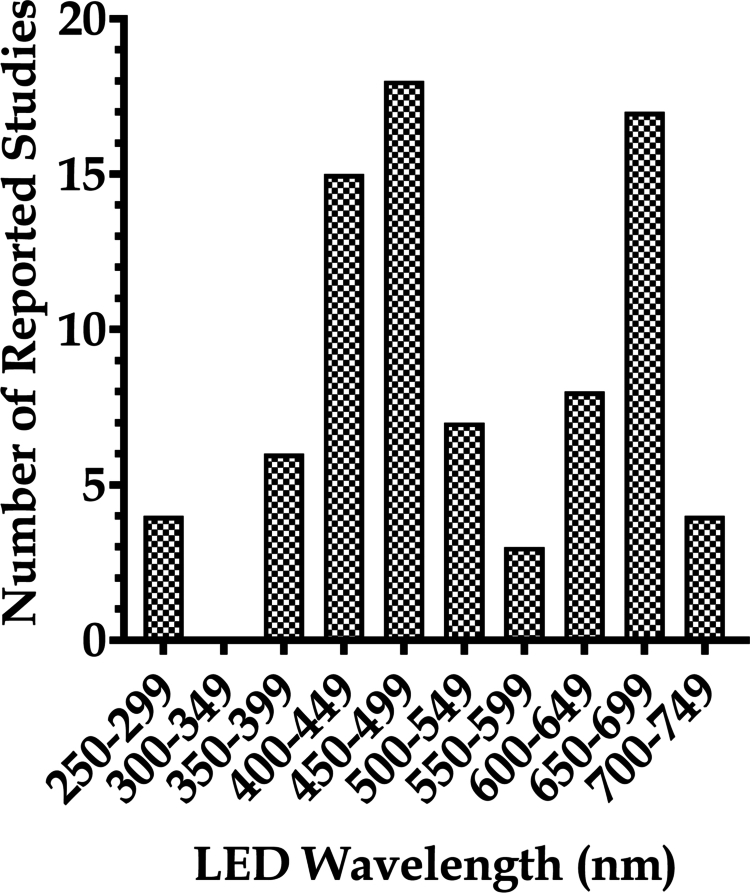
Frequency distribution of LED wavelengths (nm) used across selected studies. The wavelengths were grouped into 50 nm intervals (e.g., 250–299, 300–349, etc.), and the frequency represents the number of studies reporting LED use within each range. This analysis includes only those studies that explicitly reported exact LED wavelengths (in nm). Studies that used mixed or broad-spectrum lighting without specifying exact wavelengths, as well as those that described light only in terms of color (e.g., red, blue) without numerical values, were excluded from this dataset. Therefore, the corresponding bar graph represents the distribution of precisely defined LED wavelengths used in the selected studies.

This review addresses these gaps by providing a comparative and integrative analysis of LED and UV applications across the preharvest–postharvest continuum, and specified LED wavelengths are also quantitatively represented in Figure 1. In contrast to previous reviews, this work is structured to align physiological, biochemical, and molecular responses with wavelength-specific effects across different commodities and developmental stages. Specifically, this review (1) synthesizes evidence on how spectral quality regulates fruit growth, metabolism, and ripening processes, (2) examines the wavelength-dependent modulation of antioxidant systems, bioactive compounds, and gene expression, (3) evaluates the postharvest applications of LED and UV treatments in relation to shelf-life extension, microbial control, and senescence delay, and (4) analyzes sources of variability, methodological limitations, and challenges in translating laboratory findings to commercial systems.

**Figure 2.**
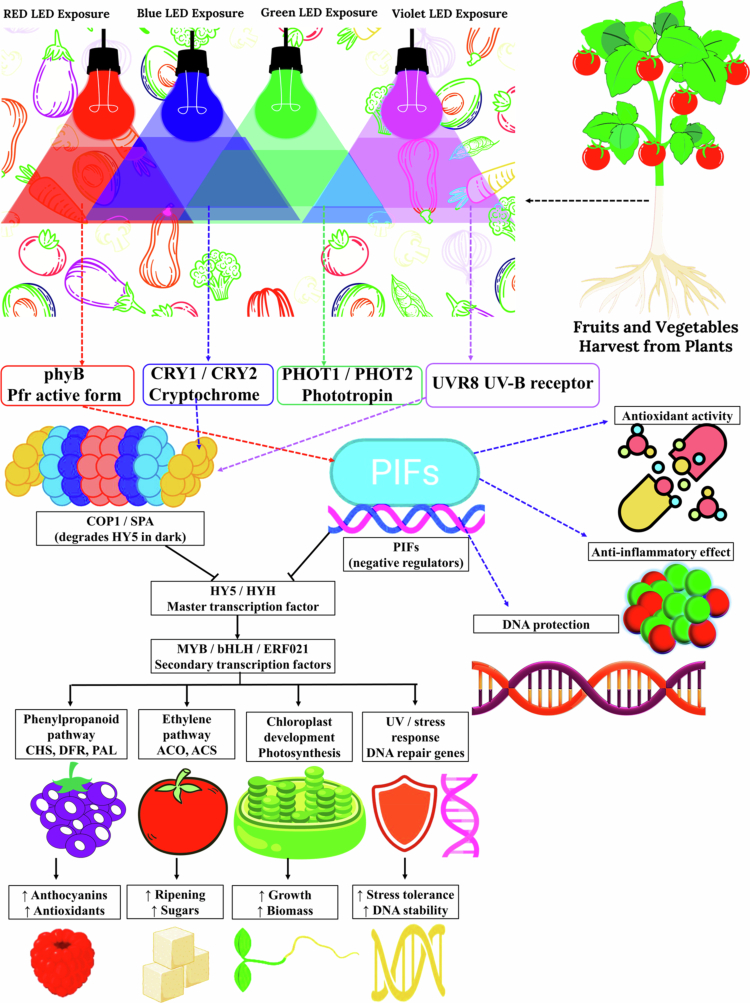
Mechanistic overview of plant perception, signaling, and physiological responses to LED and UV radiation. Different spectral regions of light, including red, blue, green, and ultraviolet wavelengths, are perceived by specific photoreceptors (e.g., phytochromes, cryptochromes, phototropins, and UVR8). These photoreceptors activate downstream signaling cascades involving key transcriptional regulators, such as phytochrome-interacting factors (PIFs) and HY5, which modulate gene expression and metabolic pathways. The integration of light signaling with hormonal regulation, redox balance, and secondary metabolite biosynthesis leads to coordinated physiological responses, including the modulation of photosynthesis, antioxidant accumulation, pigment synthesis, fruit ripening, and stress resistance. These processes collectively determine postharvest quality attributes such as nutritional value, shelf-life, and resistance to microbial spoilage. Image created with www.biorender.com.

By integrating findings across multiple levels, from physiological responses to molecular regulation and practical implementation, this review aims to provide a more coherent understanding of light-mediated effects in horticultural systems. Rather than presenting descriptive summaries alone, it emphasizes cross-study comparisons, the identification of inconsistencies, and the need for standardized experimental frameworks. A schematic overview of the key mechanisms underlying plant light perception, signaling, and downstream physiological responses to LED and UV radiation is presented in Figure 2, which illustrates the connections between photoreceptor activation, hormonal regulation, redox balance, and metabolic outcomes. Overall, this work seeks to clarify current knowledge gaps and support the development of more predictive, context-specific, and potentially scalable light-based strategies for sustainable postharvest management.

## Methodology

### Literature search and study selection

A structured and systematic literature search was conducted to identify relevant studies examining the application of light-emitting diode (LED) and ultraviolet (UV) technologies in preharvest and postharvest systems of fruits and vegetables. Major scientific databases, including PubMed, Scopus, and Google Scholar, were used to ensure comprehensive coverage of peer-reviewed literature.

### Search strategy and keywords

Search queries were developed using combinations of controlled vocabulary and free-text terms to capture interdisciplinary research. Keywords included: “light-emitting diodes (LEDs),” “UV radiation,” “postharvest preservation,” “fruit ripening,” “senescence,” “antioxidants,” “bioactive compounds,” “microbial inactivation,” “shelf-life,” “horticultural crops,” “photoperiod,” and “light quality.” Boolean operators (AND/OR) were applied to refine search outputs and improve relevance.

### Time frame

The review includes studies published between 2020 and 2026, encompassing approximately 5 y of advancements in LED and UV technologies and their applications in horticultural systems.

### Language and publication type

Only peer-reviewed articles published in English were included to ensure consistency in interpretation, quality of evidence, and accessibility.

### Inclusion criteria

Studies were included if they:

- Investigated LED or UV applications in preharvest or postharvest systems of fruits and vegetables.
- Reported experimental, field-based, or applied outcomes related to ripening, senescence, microbial control, antioxidant activity, or quality attributes.
- Included *in vivo, in vitro*, or storage-based studies relevant to horticultural commodities.

### Exclusion criteria

Studies were excluded if they:

- Were not published in English.
- Consisted only of abstracts, conference proceedings, editorials, or commentaries.
- Did not explicitly involve light-based (LED/UV) interventions.
- Focused solely on non-horticultural systems without translational relevance.

### Screening and data extraction

All retrieved records underwent a two-stage screening process:

- Title and abstract screening.
- Full-text eligibility assessment.
- From each eligible study, the following data were extracted:
- Application context (preharvest, postharvest, or combined systems).
- Light characteristics (wavelength, intensity, spectral composition, exposure duration).
- Experimental conditions (temperature, storage conditions, crop type).
- Reported outcomes (e.g., ripening rate, antioxidant levels, microbial reduction, shelf-life extension).
- Reference management and verification were conducted using Mendeley to ensure accuracy and consistency.

### Data synthesis and evidence structuring

To address heterogeneity across studies, extracted data were organized into the following thematic domains:

- Light regulation of fruit growth and development.
- Spectral effects on metabolism and molecular regulation.
- Postharvest shelf-life extension and senescence delay.
- Enhancement of bioactive compounds and nutritional quality.
- Light-mediated control of ripening and energy metabolism.
- Microbial safety, preservation technologies, and quality monitoring.
- Challenges, limitations, and translational considerations.
- Findings were synthesized using a comparative evidence-based approach.
- Structured summary tables highlighting wavelength-specific responses.
- Cross-study comparisons of physiological, biochemical, and molecular outcomes.

### Quantitative evidence mapping

Due to variability in experimental design and reporting formats, a formal meta-analysis was not feasible. Instead, a quantitative evidence mapping approach was adopted, as shown in Figure 1.

- Studies reporting explicit wavelength data were compiled
- The distribution of wavelengths across studies was visualized (e.g., frequency-based plots)
- This approach provides an overview of spectral usage trends without overinterpreting effect sizes

### Handling of heterogeneity

Variability across studies (e.g., species, cultivar, developmental stage, light intensity, storage conditions) was addressed through:

- Thematic categorization.
- Context-specific comparison within similar experimental frameworks.
- Identification of species-specific and environment-dependent responses.

### Limitations

This review is subject to several limitations:

- Inconsistent reporting of light parameters (wavelength, irradiance, duration).
- Lack of standardized outcome metrics across studies.
- Limited long-term and commercial-scale validation.
- Potential publication bias toward positive results.
- Additionally, only studies with sufficient methodological detail were included in comparative synthesis and quantitative mapping. The heterogeneity of experimental designs limits direct comparability but reflects the current state of the field.

## LED light regulation of fruit growth and development

**Figure 3.**
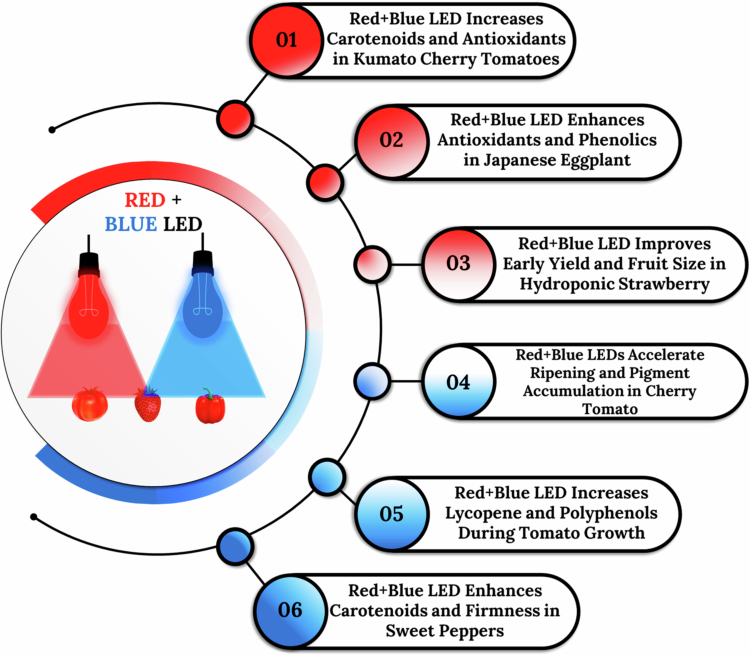
Red–blue spectral combinations are consistently associated with enhanced carotenoid accumulation, increased antioxidant and phenolic content, improved fruit size and early yield, and accelerated ripening processes across multiple crops, including tomato, strawberry, eggplant, and sweet pepper. These responses reflect coordinated regulation of primary and secondary metabolism, although the magnitude and direction of effects vary depending on the species, cultivar, developmental stage, and experimental conditions. Image created with www.biorender.com.

**Figure 4.**
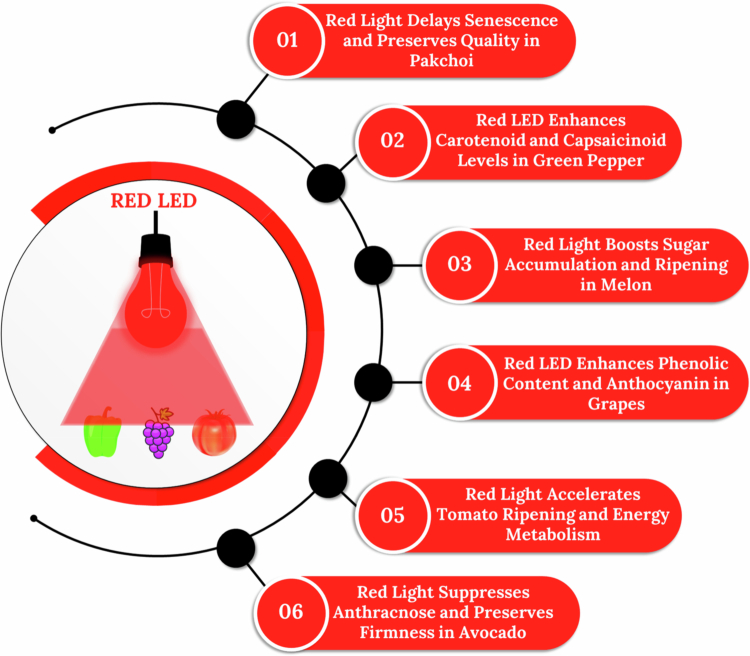
Overview of physiological and metabolic responses to red LED illumination in fruits and vegetables. Red light is primarily associated with accelerated ripening, increased sugar accumulation, enhanced carotenoid and phenolic content, and the modulation of energy metabolism. In postharvest systems, red LED treatment also contributes to delayed senescence and reduced disease incidence in crops such as pakchoi and avocado. These findings highlight the role of red light in regulating phytochrome-mediated pathways and ripening-associated processes across diverse horticultural commodities. Image created with www.biorender.com.

**Figure 5.**
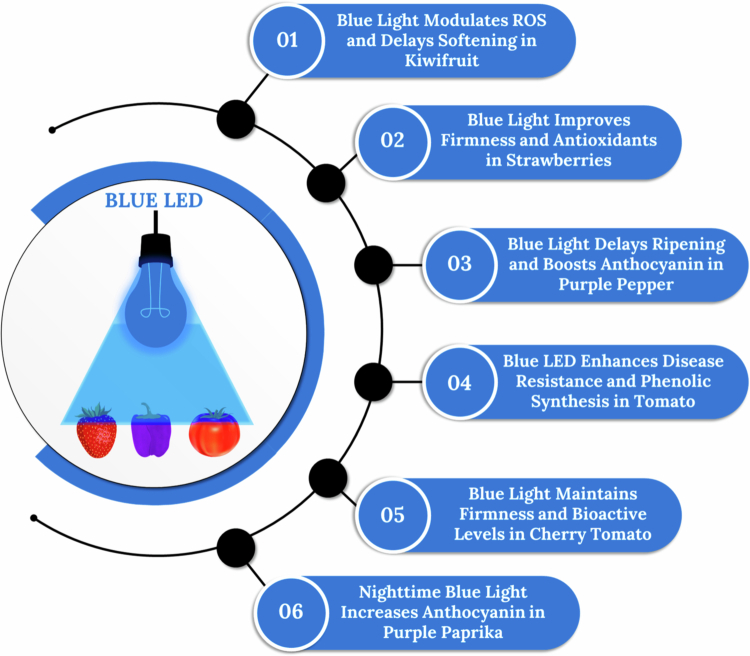
The effects of blue LED illumination on fruit and vegetable quality attributes are consistently linked to enhanced antioxidant capacity, increased phenolic and anthocyanin accumulation, improved firmness, and delayed ripening or softening in crops such as kiwifruit, strawberry, tomato, and pepper. These responses are largely associated with the activation of secondary metabolic pathways and redox regulation mechanisms, although effects on growth and yield are generally limited and highly context-dependent. Image created with www.biorender.com.

**Figure 6.**
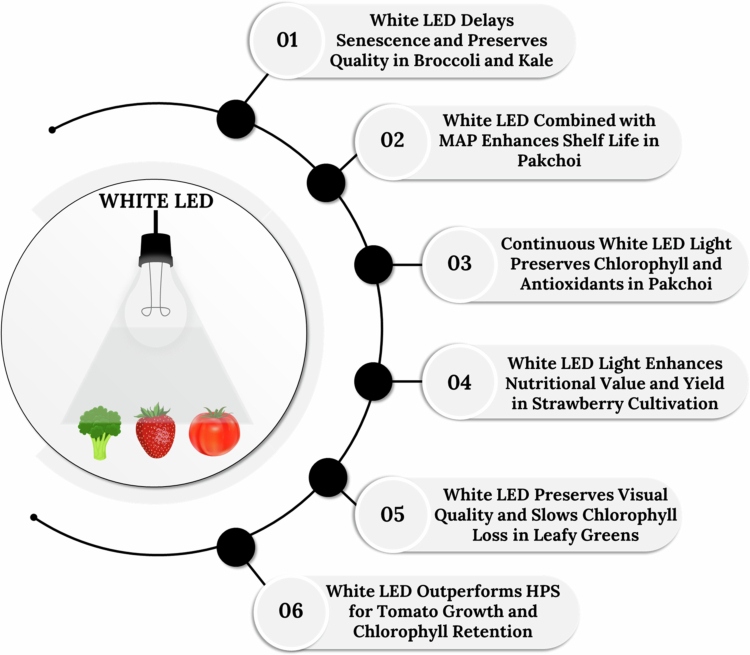
Consolidated effects of white LED illumination on the postharvest quality and shelf-life of fruits and vegetables. White light, either alone or in combination with modified atmosphere packaging (MAP), is associated with delayed senescence, preservation of chlorophyll and antioxidant compounds, and improved visual and nutritional quality in leafy vegetables and fruits. These effects are primarily linked to the maintenance of cellular integrity, reduced oxidative stress, and the modulation of respiration and degradation pathways. Image created with www.biorender.com.

**Figure 7.**
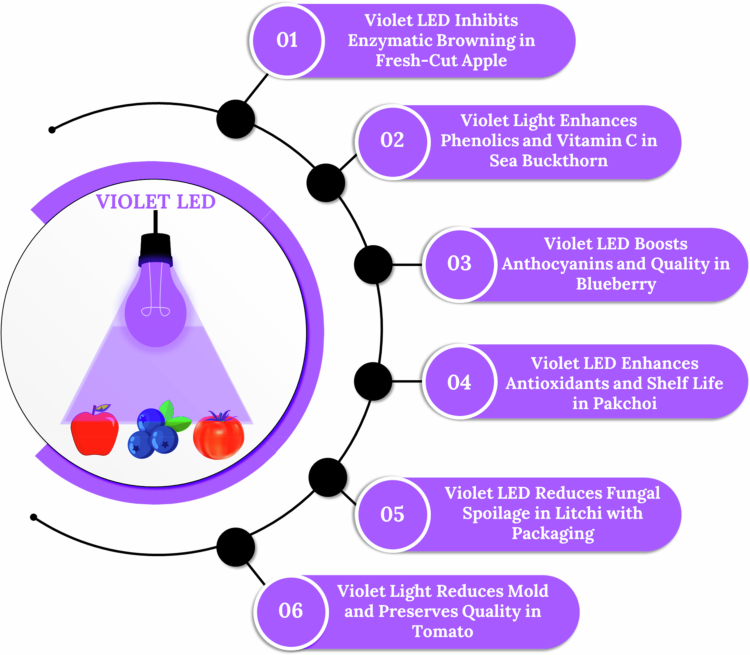
Violet LED (short-wavelength) effects on postharvest quality and metabolic responses in fruits and vegetables. Violet light is associated with the inhibition of enzymatic browning, increased phenolic and vitamin C content, enhanced anthocyanin accumulation, and reduced fungal spoilage in crops such as apple, blueberry, tomato, and litchi. These responses are linked to the activation of phenylpropanoid pathways and the suppression of oxidative enzymes, highlighting the role of high-energy wavelengths in regulating stress-related metabolic processes. Image created with www.biorender.com.

Light-emitting diode (LED) technology enables precise manipulation of light quality in horticultural systems, allowing detailed investigation of how specific wavelengths regulate fruit growth, development, and composition. Each wavelength exerts distinct effects on plant physiology, and their applications are highlighted in Figures 3–7, while a comparative synthesis of wavelength-specific responses across major fruit crops is presented in Table 1. Studies across strawberry, tomato, pepper, paprika, passion fruit, microtomato, and hot pepper collectively show that red, blue, far-red, green, and ultraviolet light influence carbohydrate allocation, organic acid metabolism, pigment synthesis, antioxidant accumulation, and ripening, often independently of overall vegetative growth. As summarized in Table 1, spectral combinations modulate key processes such as carbon partitioning, sink strength, and metabolic regulation through coordinated physiological and biochemical pathways, with responses strongly shaped by species, cultivar, developmental stage, and cultivation conditions.

**Table 1.** Wavelength-specific effects of LED light treatments on fruit growth, metabolism, and quality in horticultural crops. The table summarizes the effects of spectral composition on carbon partitioning, sink strength, ripening, and antioxidant accumulation, with mechanistic insights provided where supported by evidence. Arrows (↑, ↓) indicate relative increases and decreases compared with control or reference conditions.

Crop	Light treatment	Key effects	Mechanistic insight	Reference
Strawberry (*Fragaria *×* ananassa* Duch.)	Red–blue LEDs (2:1, 1:2) + MeJA/MeSA	↓ glucose & fructose; ↑ malic acid	Carbon allocation shifts toward defense-related metabolism under LED–hormone interaction	[17]
Tomato (*Solanum lycopersicum*)	Blue–violet LED (450–485 nm); induction vs HPSL	Blue: ↑ phenolics, ↑ β-carotene; HPSL: ↑ soluble solids	Blue light promotes secondary metabolism; red/HPSL favors primary metabolism	[18]
Tomato (*Solanum lycopersicum*, cv. Moneymaker)	Red–blue + far-red (FR)	↑ sink strength; ↑ sugars (starch, sucrose, glucose, fructose)	FR enhances sugar transport and carbon partitioning rather than photosynthesis	[19]
Sweet pepper (*Capsicum annuum* L.)	Red–blue vs red–blue + FR (8 R:2B)	RBFR: ↑ yield, ↓ carotenoids; RB: moderate yield, higher carotenoids	FR improves light penetration and sink strength but induces a yield–quality trade-off	[20]
Passion fruit seedlings (*Passiflora edulis*)	Red, green, blue LEDs (varying ratios/intensity)	↑ growth, biomass, chlorophyll; ↑ antioxidants (blue-enriched spectra); low intensity ↓ performance	Blue light enhances photosynthesis and secondary metabolism; an adequate intensity is required	[21]
Tomato (*Solanum lycopersicum* L.)	Blue (480 nm) & red (660 nm) ± UV-B	Blue + UV-B: ↑ photosynthesis, ↑ flavonoids; Red: ↑ respiration, photoprotection	UV-B induces phenolics; blue supports photosynthesis; red increases carbon loss	[22]
Tomato (*Solanum lycopersicum*)	Red (634 nm) or blue (450 nm), pre-/postharvest	↑ lycopene & β-carotene; accelerated ripening	Direct fruit illumination stimulates ripening processes	[23]
Dwarf tomato (*Solanum lycopersicum* L. *cv. Micro Tom*)	Red (660 nm), blue (447 nm), FR (740 nm), green (523 nm) and UV-A (385 nm)	No significant effect on growth or yield	Light effects are stage-dependent, with the strongest responses during fruit maturation	[24]
Hot pepper (*Capsicum annuum* L.)	4 R:1B:5 W LED combination (Red 700 nm, and Blue 465 nm)	↑ ripening, seed development, flavonoids, organic acids, ↓ amino acids, carbohydrates	Light regulates phenylpropanoid metabolism via ERF021 and shifts carbon allocation	[25]

Across these crops, one recurring theme is that short-wavelength–enriched spectra tend to shift carbon towards secondary metabolism and stress‑related pathways, whereas red and far‑red light more strongly modulates sink strength, fruit growth, and ripening dynamics. Adrian et al. exemplified this secondary‑metabolism–oriented response in strawberry grown under lowland tropical conditions using a two‑factor randomized block design that combined red–blue LED ratios (2:1, 1:2, and 1:1) with exogenous methyl jasmonate (MeJA) and methyl salicylate (MeSA) at four concentrations. By integrating GC‑MS and LC‑MS with conventional physiological measurements, they showed that LED–hormone combinations triggered defense‑related secondary metabolites, terpenoids, alkaloids, saturated fatty acids, and amino acid derivatives, even when shoot growth, chlorophyll content, and fruit TDS/TTA were statistically unchanged, highlighting a dissociation between visible plant physiology and underlying biochemical shifts. Leaf glucose and fructose accumulated 27%–64% less in the LED–hormone treatments than in the controls, which the authors interpreted as accelerated mobilization of photosynthates towards reproductive sinks, although this remains mechanistically under‑substantiated without flux or enzyme‑level data. High MeJA (15 mM) inhibited flowering, presumably by suppressing photosynthesis and constraining assimilate availability, whereas equivalent MeSA concentrations still allowed fruiting, indicating that distinct hormonal modes of action captured observationally but not fully mechanistically resolved; concomitant increases in malic acid (5.76 mg mL^−1^ under MeJA CJ1; 5.91 mg mL^−1^ under MeSA BS3) point to altered organic acid metabolism with potential implications for fruit flavor that were not evaluated sensorially. Multivariate analyses further revealed that hormone type, rather than the LED spectrum, was the dominant driver of secondary metabolite clustering, raising questions about the statistical power to detect subtle LED‑specific metabolomic effects given that only three composite replicates per treatment were analyzed.^17^

Tomato studies deepen this distinction between primary and secondary metabolism and underscore strong cultivar‑specific interactions with light quality. Alsina et al. conducted a three‑year greenhouse experiment comparing LEDs, induction lamps, and high‑pressure sodium lamps (HPSL) across five tomato cultivars, thereby providing unusually robust longitudinal evidence. Rather than a single superior light source, they found pronounced cultivar × lighting interactions: LED lighting increased β‑carotene in “Chocomate” by 34.3% but reduced it in “Bolzano” by 18.5%, while “Encore” and “Strabena” were largely unresponsive to spectral changes. HPSL consistently produced the highest dry matter and soluble solids (4.7%–18.2% above LED and induction), but this was confounded by slightly higher lamp‑level temperatures that could have concentrated solutes through mild water stress rather than purely photobiochemical effects. The authors hypothesized that the ~20% blue–violet content in LED and induction lighting drove 1.6%–47.4% higher phenolic levels relative to HPSL, which was consistent with the blue/UV activation of phenylpropanoid pathways, yet statistically significant phenolic differences appeared only sporadically (e.g., flavonoids in “Chocomate”), reflecting high within‑treatment variability linked to shifting ripening stages across the November–March harvest window. Their non‑parametric analysis (Kruskal‒Wallis and Wilcoxon) was appropriate for non‑normal data, but the nested structure of repeated harvests within seasons and years was not modeled explicitly, likely inflating residual variance and obscuring some treatment effects. Even so, this study strongly supports the practical conclusion that supplemental light source selection should be cultivar‑specific rather than universally favoring LEDs over HPSL.^18^

Within tomato, far‑red (FR) light has emerged as a key regulator of sink strength and dry‑matter partitioning. Ji et al. provided one of the most mechanistically complete demonstrations of this effect by combining greenhouse trials, a LINTUL‑based crop partitioning model, carbohydrate profiling, and RT‑qPCR. FR radiation increased individual tomato fruit sink strength by 38%, and when this measured increase was incorporated into the crop model, it quantitatively reproduced the observed rise in the fruit dry‑mass fraction without invoking changes in vegetative sink strength, an unusually strong mechanistic link from light quality to whole‑plant carbon allocation. Trusses were pruned to a single fruit to isolate potential growth, and the authors validated this design choice by showing no growth rate difference between one‑fruit and two‑fruit trusses. At the molecular level, FR upregulated sugar transporter genes (SUTs, HTs, and SWEETs) around anthesis, then sucrose synthase (SUS1) and invertase (LIN7) at 10–20 d after anthesis (DAA), followed by broader activation of sucrose cleavage and starch catabolism genes at 30 DAA, mapping onto the known transition from starch to hexose accumulation in developing tomato fruit. However, concurrent upregulation of INVINH1, an inhibitor of the cell‑wall invertase LIN5, introduced a mechanistic paradox that the authors interpreted as fine‑tuning but did not resolve with enzyme activity measurements; similarly, unchanged sucrose concentrations were explained as evidence of increased import to match higher demand, an inference not independently supported by phloem flux data. Differences in CO₂ partial pressure between the greenhouse (680 µbar) and climate‑chamber experiments (488 µbar) also introduce an environmental confound that was not fully addressed. Despite these limitations, Ji et al. effectively linked a specific light quality (FR) to a mechanistic chain spanning gene expression, carbohydrate metabolism, organ‑level sink strength, and whole‑plant partitioning, providing a firm basis for FR‑supplemented LED strategies in greenhouse tomato.^19^

Sweet pepper studies revealed that this FR‑mediated enhancement of sink strength and yield often entails a trade‑off with fruit quality, particularly carotenoid accumulation. Kim and Son evaluated red–blue (RB) LED interlighting with or without FR supplementation in greenhouse sweet pepper and showed that RBFR increased total fruit yields by 9% (red fruits) and 19% (yellow fruits) compared with RB alone, yet reduced total carotenoids by ~18%–24%. Their two‑group sampling design, in which Group 1 developed under mainly natural light (interlighting ~13% of the daily light integral, DLI) and Group 2 under a near‑equal natural: artificial ratio (~40%), demonstrated that the magnitude and significance of spectral effects are strongly conditioned by the seasonal background DLI, with Group 2 exhibiting more pronounced and statistically robust responses. The carotenoid reduction under RBFR was mechanistically attributed to a lower R:FR ratio (0.91), maintaining more phytochrome in the inactive Pr form, thereby sustaining high PIF1a levels that suppress PSY1 expression; while this explanation is consistent with tomato literature, the study did not measure the PSY1, PIF1a, or phytochrome photostationary state at the fruit level, leaving the mechanism is inferential. Interestingly, the ascorbic acid content was higher under RBFR than RB in Group 2 (by ~9% in red and 3%–4% in yellow fruits), suggesting that, in an interlighting context where FR supplements rather than replace PAR, photosynthetic enhancement may offset spectral trends that otherwise decrease ascorbate. RB alone reduced individual fruit weight compared with both natural light and RBFR, a result attributed to altered R:FR environments within the canopy interior where FR reflected or scattered from upper leaves is lacking. With three replicates per treatment (*n* = 4–5 for quality traits) and no multi-season replication, some seasonal and developmental effects remain confounded, but this work clearly documents an FR‑driven yield–carotenoid trade‑off that forces growers to prioritize either yield or carotenoid‑rich functional quality.^20^

Beyond solanaceous crops, blue‑enriched spectra also emerge as particularly influential in seedling development and antioxidant metabolism, but their effects are strongly context‑dependent. Liang et al. used an orthogonal L9(3³) design in passion fruit (*Passiflora edulis*) seedlings to test light irradiance (50, 100, and 200  µmol/m^−2^ s^−1^), spectral ratios (R70:B30, R30:B70, and R50:G20:B30), and photoperiods (10/14 h, 12/12 h, and 14/10 h) across nine LED combinations. Range analysis identified LM5 (100 µmol/m^−2^ s^−1^, R30:B70, 14 h/10 h) as optimal, with the highest plant height (69.0 cm), stem diameter (4.11 mm), leaf number (12.4), internode length (71.5 mm), and shoot and root dry mass, which is consistent with blue‑light enhancement of growth in this species. However, the large internode distance under LM5 may reflect elongation responses that border on etiolation, a nuance not discussed by the authors. Irradiance (factor A) was consistently the most influential driver of growth and pigment traits, confirming that photon quantity dominated over spectral quality within the tested range and raises questions about whether these spectral responses would persist at saturating light levels. The antioxidant enzyme patterns introduced a further complexity: LM5 produced the highest peroxidase activity and total phenol and flavonoid contents, which are typically interpreted as stress markers, yet also supported maximal biomass, making it unclear whether these responses reflect productive secondary metabolism or co‑occurring mild stress, an ambiguity that cannot be resolved without ROS or additional stress indicators. Catalase activity peaked in LM1 (50 µmol m^−2^ s^−1^, R70:B30, 10/14 h) and was not significantly affected by irradiance, while POD and SOD showed different optima, confirming that antioxidant enzymes responded via distinct regulatory pathways and cannot be collapsed into a single “antioxidant status” metric. Comparison with a white, fluorescent control (100 µmol m^−2^ s^−1^, 12/12 h) was constrained by incomplete spectral characterization of the control and its ambiguous positioning within the orthogonal design, and the study's four biological replicates and 60‑d seedling window limit generalization to full production cycles.^21^

In vertical‑farming microtomato, blue and UV‑B light also jointly optimize photosynthesis and flavonoid accumulation, again illustrating that some spectral regimes can simultaneously support growth and defense. Lima et al. tested six LED treatments (RBW, RBW + UV, B, B + UV, R, R + UV at 300 µmol m^−2^ s^−1^) in a completely randomized design and showed that monochromatic blue light supplemented with UV‑B (BUV) produced the highest photosynthetic rate (A = 11.57 µmol m^−2^ s^−1^), stomatal conductance, and rutin concentrations across three fruit maturation stages. Green fruits under B reached 853.14 mg kg^−1^ rutin, and orange fruits under BUV reached 882.06 mg kg^−1^, whereas R and R + UV yielded the lowest rutin levels (68.23 and 27.36 mg kg^−1^), implying ~12–32‑fold differences driven by spectral quality. The authors propose that blue light activates cryptochrome/phototropin signaling to enhance CO₂ diffusion and phenylpropanoid biosynthesis, while UV‑B engages UVR8‑mediated pathways; however, no gene expression data (CRY, PHOT, UVR8, PAL, and CHS) were provided, so mechanisms remain to be inferred. The lethal effect of R + UV, where plants died, and no fruit data were obtained, underscores that UV‑B tolerance is strongly dependent on the spectral context, with blue light apparently pre‑conditioning photoprotective responses that red light alone cannot support, although this potentially important interaction has not been fully explored. The red‑only plants showed a coherent stress phenotype: elevated nocturnal respiration, low Fv/Fm, high unregulated energy dissipation (YNO), and low ETR and qP, which was consistent with PSII dysfunction under monochromatic red. RBW + UV produced unexpectedly low stomatal conductance, low photosynthesis, high ETR/A, and low rutin, suggesting complex interactions among spectral components and UV dose fractionation that were not dissected. Non‑parametric statistics with FDR correction and PCA appropriately summarized multivariate patterns, but the single 40‑d run and six replicates per treatment limit power and exclude chamber‑to‑chamber or temporal replication. Rutin quantification was methodologically robust (HPLC, *R*² = 0.9999), though whole‑fruit analysis without peel–pulp separation constrains comparison with peel‑enriched polyphenol datasets.^22^

Postharvest and stage‑specific studies further demonstrated that developmental timing often modulates responsiveness to LED spectra as strongly as the wavelength itself. Ngcobo et al. tested 48‑h red or blue LED treatments at the green‑mature and turning stages in two cherry tomato cultivars and found that green‑mature fruit responded far more strongly to both wavelengths, with greater changes in a* and b* color coordinates, lycopene, β‑carotene, chlorophyll degradation, and total soluble solids (TSS) than turning‑stage fruit. This suggests that physiology at earlier ripening stages is more amenable to spectral manipulation, which is consistent with the fact that the chloroplast‑to‑chromoplast transition being fully initiated in green‑mature tissue. Red light more strongly enhanced lycopene in the red cultivar, while blue light preferentially increased β‑carotene in both cultivars, a pattern plausibly explained by phytochrome‑ and cryptochrome‑mediated regulation of PSY1 and LCYB, respectively, but no phytochrome photostationary state, ethylene, or gene expression data were collected. Firmness was assessed using a subjective three‑category “finger feel” scale and analyzed by ANOVA as if continuous, an approach that likely reduced sensitivity to softening differences. TSS increases were significant at the green‑mature stage, but mechanistic claims about sugar‑specific changes remained speculative in the absence of individual glucose, fructose, and sucrose measurements. With three replicates, a single glasshouse environment, and no white‑light or mixed spectral control, wavelength effects cannot be fully separated from broader illumination differences, yet the study still provides applied evidence that short, commercially feasible LED exposures can advance color and carotenoid development without compromising mass or firmness.^23^

The temporal dimension of spectral responses was further explored by Samuolienė et al., who examined whether dynamic, stage‑specific spectral sequences outperform constant spectra across the seedling, vegetative, and fruiting stages in Micro Tom tomato. Using three spectra, S (red/blue/far‑red), G (red/blue/far‑red/green), and F (red/blue/far‑red/UV‑A), applied in thirteen combinations at 250 µmol m^−2^ s^−1^, they found that plant performance was predominantly determined by the spectrum applied during the current growth stage rather than the cumulative spectral history, with treatments converging in multivariate space according to the final‑stage spectrum. An exception was mineral nutrition, where green‑enriched G spectra increased macro- and micro‑element contents (1.2–1.6 × higher K, Mg, P, Ca, Fe, Zn, Mn, and Cu compared with S), suggesting that nutrient accumulation may be more sensitive to spectral sequencing than to gross morphology or photosynthesis. UV‑A‑enriched F spectra induced mild photoinhibition in seedlings (Fv/Fm ≈ 0.73) yet enhanced seedling biomass and, later, photosynthesis, water‑use efficiency, and light‑use efficiency, implying a growth‑promoting but stage‑dependent role for UV‑A. The study's use of a dwarf determinate cultivar constrains generalizability to commercial indeterminate tomatoes, and secondary metabolites have not been evaluated, leaving open how dynamic spectra affect lycopene or phenolic profiles, but the findings challenge assumptions that complex lighting recipes necessarily outperform simpler, final‑stage‑focused designs.^24^

Finally, hot pepper work illustrates how mixed‑spectrum LEDs can be tuned to accelerate reproductive development while reshaping metabolic allocation. Zheng et al. used multi‑omics to evaluate red/blue/white LED ratios in *Capsicum annuum* and identified 4R:1B:5W as uniquely effective at shortening the time to full ripening by ≥16 d, increasing the proportion of red fruits to 14.48% (vs. 3.85% under white), and boosting yield per plant and individual fruit mass. Developmental staging revealed that the seed‑to‑first‑flower phase (S1) was rate‑limiting, with 4R:1B:5W compressing this interval while 7 R:1B:2 W prolonged it, suggesting that excessive red light suppresses reproductive transition, likely via phytochrome‑mediated CO and FT repression. Metabolomics showed that 4R:1B:5W elevated flavonoids (e.g., vitexin, cyanin), organic acids, and phenolics while reducing amino acids and carbohydrates, indicating a reallocation from primary to secondary metabolism; transcriptomics implicated ERF021 as a central hub linking light signaling to these metabolic shifts. However, the expected ERF‑linked upregulation of amino acids and carbohydrates did not materialize, prompting the authors to speculate that high‑light‑induced ROS may disrupt primary metabolism, an interpretation that remains untested. The 7R:1B:2W treatment provided a useful negative reference, inducing dark‑purple, morphologically abnormal flowers and broad metabolite suppression, emphasizing the non‑linear, threshold‑dependent nature of red‑light responses. Conducted in a single cultivar under controlled hydroponic conditions, this work nonetheless proposes a concrete spectral “recipe” (4R:1B:5W) and nominates ERF021 as a candidate regulator for future functional studies.^25^

Taken together, these studies show that LED spectral composition regulates fruit growth and development through integrated effects on carbon partitioning, sink strength, metabolite allocation, and developmental timing, but they also expose key limitations that complicate cross‑study synthesis. The blue‑ and UV‑enriched spectra frequently promote secondary metabolism and antioxidant accumulation in strawberry, passion fruit, and microtomato, yet their impact on biomass ranges from neutral to strongly positive, depending on irradiance, photoperiod, and developmental stage, indicating that “high‑blue” lighting cannot be assumed to have uniform growth outcomes across species or production systems. Far‑red supplementation consistently increases sink strength and yield in tomato and sweet pepper, but this benefit is often accompanied by dilution of carotenoids and other quality traits, underscoring an inherent trade‑off between productivity and nutritional density that needs explicit consideration in commercial lighting design. Stage‑specific and cultivar‑specific responses are pervasive, for example, green‑mature versus turning‑stage tomatoes, or strongly photo‑responsive versus largely insensitive tomato and pepper genotypes, demonstrating that universal spectral prescriptions are premature and that cultivar‑ and stage‑tailored recipes are likely to be more effective than “one‑size‑fits‑all” solutions. At the same time, methodological constraints, including small sample sizes, orthogonal rather than full‑factorial designs, reliance on single cultivars, short experimental windows, and limited use of flux measurements, enzyme assays, or gene‑level validation, restrict generalizability and make it difficult to disentangle genuine biological divergence from context‑dependent artefacts. Addressing these gaps will require longitudinal, multi‑cultivar studies that integrate controlled spectral treatments with detailed physiological and multi‑omics analyses, ideally across contrasting production environments, to quantify yield–quality trade‑offs and identify robust, crop‑specific spectral regimes for LED‑based regulation of fruit growth and development.

## Spectral light effects on photosynthesis, metabolism, and molecular regulation

Light quality is a key regulator of plant metabolic pathways that determine nutritional composition, pigment accumulation, antioxidant capacity, and postharvest quality. With the increasing use of LED systems in controlled environments, research has moved toward understanding how specific wavelengths regulate transcriptional, metabolic, and hormonal processes. A comprehensive overview of these wavelength-dependent effects, including mechanistic interpretations across horticultural crops, is provided in Table 2. Together, the following studies show how blue, red, violet, and composite spectra influence biosynthetic pathways, revealing both conserved regulatory patterns and marked context dependence across species, tissues, and developmental stages.

**Table 2.** Effects of LED spectra on crop metabolism, development, and postharvest responses, including key mechanisms. Arrows (↑, ↓) indicate increases or decreases relative to controls.

Crop	Light treatment	Key effects	Mechanistic insight	Reference
Pakchoi (*Brassica rapa ssp. chinensis “Black Behi”*)	Blue–white, red–white, white LEDs	Blue–white: ↑ carotenoids (~15%); Red–white: ↓ carotenoids	Blue light enhances carotenoid biosynthesis at the transcriptional level	[26]
Tomato (*Solanum lycopersicum* L. *“Micro-Tom”*)	Supplemental blue light (430 nm; pulsed S10 optimal)	↑ flowering, ripening, ethylene, lycopene, antioxidants	Pulsed blue light promotes ethylene biosynthesis, coordinating fruit ripening and metabolite accumulation	[27]
Purple paprika (*Capsicum annuum* “Tequila”)	Night-time blue light (460 nm)	↑ anthocyanins (no change in fruit size)	Blue light activates the HY5–MYB–bHLH pathway for anthocyanin synthesis	[28]
Blueberry (*Vaccinium corymbosum* “Star”)	Blue (460 nm), red (660 nm), yellow (590 nm), white light	Blue/white: ↑ fruit size; Blue: ↑ anthocyanins; Red/yellow: ↑ antioxidant enzymes	Light quality drives metabolic allocation toward anthocyanins or antioxidant systems	[29]
Tomato (*Solanum lycopersicum*)	Blue (470 nm) vs red (655 nm)	Blue: ↑ amino acids, secondary metabolites; Red: altered ion profile	Blue and red light regulate distinct metabolic and ion transport pathways	[30]
Grape (*Vitis vinifera* “Cabernet Sauvignon”)	Blue (450 nm), red (660 nm)	Blue: ↑ anthocyanins, sugars; Red: opposite effects	HY5/MYB vs PIF-mediated pathways drive contrasting metabolic responses	[31]
Oriental melon (*Cucumis melo* var. *makuwa*)	Red light (660 nm)	↑ ethylene and sucrose during ripening	Red light promotes ethylene synthesis via PIF regulation	[32]
Apple (*Malus *×* domestica*)	Violet light (433 nm)	↓ browning; ↑ phenolics	HY5-related regulation enhances phenolic synthesis and reduces browning	[33]
Kiwifruit (*Actinidia chinensis* “Jinyan”)	Blue light (420 nm)	Delayed softening; ↑ antioxidants; ↓ lipid peroxidation	Blue light enhances antioxidant systems, maintaining membrane integrity	[34]
Broccoli (*Brassica oleracea* var. *italica* “Youxiu”)	White LED + VOCs	Delayed senescence; maintained color	Combined treatment suppresses senescence-related processes	[35]

A first group of studies demonstrates that blue‑enriched spectra, often in combination with broader spectral backgrounds, act as strong drivers of carotenoid and flavonoid biosynthesis but with clear trade‑offs between nutritional quality and biomass. Frede and Baldermann address this directly in pakchoi (*Brassica rapa ssp. chinensis “Black Behi”*) sprouts by combining UHPLC‑ToF‑MS carotenoid profiling, RT‑qPCR, and a Norfurazon-based pharmacological flux assay to dissect how different LED compositions affect carotenoid biosynthesis. They show that blue + white LEDs increased total carotenoids by ~15% over white LEDs alone (baseline ~700 ng mg^−1^ DW), whereas red + white LEDs reduced carotenoids by a similar magnitude and that this increase depends on a broad-spectrum context: monochromatic blue only slightly elevated phytoene (11%–13%), while blue + white raised carotenoids by 38%–56%, indicating that elevated biosynthetic transcripts (PSY, βLCY, βOHASE1, and CCD4) translate into flux only when non‑blue wavelengths are present. The concurrent upregulation of CCD4, a carotenoid‑degrading enzyme, under blue + white implies that net carotenoid accumulation reflects biosynthesis outpacing degradation or apocarotenoid channeling rather than simple suppression of breakdown, an unresolved mechanistic nuance that the authors highlight. Practically, the same blue + white regime reduced fresh mass by ~15% relative to red + white, illustrating a clear trade‑off between nutritional enrichment and biomass driven by cryptochrome‑mediated growth suppression under high blue fractions.^26^

Building on the role of blue light in modulating both primary and secondary metabolism, He et al. extended the analysis to temporal patterns of blue‑light delivery in Micro‑Tom tomato, asking whether pulsed versus continuous blue supplementation at constant intensity (100 µmol m^−2^ s^−1^, 430 nm, 12 h d^−1^) differentially affects ripening and fruit composition. All four regimes (S6, S8, S10, S12) accelerated flowering by 1–3 d and ripening by ~3–4 d relative to natural light, but the 30‑min on/8‑min off treatment (S10) gave the earliest peak flowering and most advanced color change (lowest hue angle, highest a*/b* at 38 DAA), indicating that a moderate duty cycle is more effective than continuous exposure for synchronizing reproductive development. All blue light treatments increased ethylene production across 34–46 DAA, with S10 sustaining the highest levels and thus fitting a cryptochrome–ACO/ACS/ERF‑mediated model, although no gene expression data were collected to confirm this in the specific system. Nutritionally, lycopene, phenolics, flavonoids, vitamin C, soluble sugars, and antioxidant capacity (DPPH/FRAP) all improved under blue light, with S10 maximizing lycopene and sugars and S12 maximizing vitamin C and flavonoids, suggesting that pulse frequency and total photon dose differentially tune distinct biosynthetic pathways. This work was conducted in a dwarf cultivar under a plastic greenhouse with a variable solar background, limiting direct translation to commercial indeterminate tomatoes, but it introduces the important concept that temporal structuring of blue light can improve quality with lower total photon input than can continuous lighting.^27^

At the fruit‑surface level, Satitmunnaithum et al. show that night‑time blue light can selectively drive anthocyanin biosynthesis in colored tissues without affecting growth, extending the blue‑light narrative into purple paprika (*Capsicum annuum “Tequila”*). Using two seasonal repetitions and initiating treatment 10 d after pollination, they exposed fruit to directional blue LEDs at night and exploited the geometry of irradiation to generate directly irradiated (DR) and indirectly irradiated (IR) peel sectors on the same fruit. Across 15–40 DAP, DR peel consistently showed elevated anthocyanin contents in both seasons without any significant changes in fruit weight, length, or diameter, indicating that the blue signal was channeled into flavonoid pathway activation rather than general growth stimulation. The transcriptional data support this: upstream regulators (HY5, MYB, bHLH, WDR, and PAL) and structural genes (CHS, F3H, DFR, ANS, and UFGT) were upregulated in the DR peel, with peak expression at ~15 DAP and decline thereafter, matching the window of active anthocyanin biosynthesis. HY5 expression was numerically higher but not statistically significant, suggesting that modest HY5 induction may be amplified via its interaction with other transcription factors. Flesh tissue showed neither anthocyanin accumulation nor light‑responsive gene expression, emphasizing tissue specificity; the IR peel had intermediate responses, implying a dose–response relationship that was not fully quantified because PPFD on the IR surface was not measured. The study clearly defines a 20–40 DAP harvest window as optimal for capturing peak purple coloration, although it also shows that blue light cannot prevent later anthocyanin degradation associated with ripening‑associated metabolic reprogramming.^28^

The blueberry work of Wei et al. then demonstrated how different monochromatic wavelengths partition functions among growth, pigment accumulation, and antioxidant defense, further emphasizing that no single spectrum optimizes all traits. In potted “Star” blueberry under greenhouse conditions, blue (460 nm), red (660 nm), yellow (590 nm), and white LEDs generated distinct phenotypic profiles: blue light produced the largest, firmest berries with the highest anthocyanin content, soluble solids, solid–acid ratio, and upregulation of most anthocyanin biosynthesis genes (VcC4H, Vc4CL, VcCHI, VcDFR, and VcUFGT), whereas red light maximized SOD and POD activities, suppressed anthocyanin accumulation, and produced the smallest, most acidic fruits. Yellow light, in contrast, elevated non‑enzymatic antioxidants (AsA, GSH) and total phenolics, with VcLDOX showing peak expression under yellow rather than blue, an unexpected divergence from the otherwise blue‑dominant pattern that remains mechanistically unexplained. Pearson correlations and PCA (94.55% variance explained) support a negative relationship between enzymatic antioxidant activity (SOD/POD) and anthocyanin content under red light, leading the authors to propose that high peroxidase activity may actively degrade anthocyanins via vacuolar oxidation, a hypothesis that fits the correlations but still lacks direct enzymatic validation. The absence of mixed‑spectrum treatments is a design limitation given strong evidence elsewhere that spectral combinations outperform monochromatic regimes, but the study defines a clear blue‑light “quality optimum” for blueberry while highlighting the antagonism between pigment preservation and enzyme‑based ROS defense under red light.^29^

Moving from single pathways to integrated metabolic networks, Xiao et al. used a multi‑omics framework in Micro‑Tom tomato to dissect how monochromatic blue (470 nm) and red (655 nm) LEDs applied post‑anthesis reshaped fruit metabolism, ionome composition, and gene expression relative to white fluorescent light. Under blue light, 39.3% of the altered metabolites mapped to amino acid metabolism and 29.5% to secondary metabolism, with elevated glutamate, aspartate, GABA, citrulline, pipecolic acid, tryptamine, trigonelline, putrescine, choline, naringin, tocopherols, and total phenolics, providing a metabolite portfolio consistent with enhanced nitrogen assimilation, stress tolerance, and antioxidant capacity. Transcriptomics showed blue‑upregulated genes enriched in secondary metabolism, carbon fixation, and glycine–serine–threonine metabolism, including Calvin cycle enzymes (FBA3, FBP, GAPDH, SBP, PRK), even though some Calvin intermediates were lower, suggesting increased flux rather than accumulation. The ionomic data revealed that fruit ionomes were more responsive to spectral quality than leaves and that red light significantly increased Fe, Ni, and Rb in fruits, which was matched by the upregulation of YSL1 and MTP11 transporters, providing the first direct link between light quality and elemental redistribution in tomato fruits. Ferritin1 was upregulated under blue without a corresponding Fe increase, indicating that its role may be sequestration rather than accumulation under these conditions. Because plants were grown to flowering under white light before spectral treatments, the observed changes represent developmental responses of established plants rather than whole‑life-cycle effects, and the Micro-Tom cultivar again limits agronomic generalization, but the study convincingly demonstrated that blue and red light reorganizes whole metabolic networks and ion homeostasis, not just isolated compound classes.^30^ The use of Micro-Tom further limits generalizability for the same reasons discussed in^24^ and^27^ examined in this series. Nevertheless, multiomics integration, the first ionomic characterization of light-quality effects in tomato fruits, and gene-level mechanistic tracing of specific metabolite accumulation patterns collectively make this a methodologically sophisticated and conceptually expansive contribution to the LED horticulture literature.

A similar multi‑omics depth appears in Zhang et al., who study pre‑veraison Cabernet Sauvignon grapes under blue, red, green, and white LEDs using transcriptomics, metabolomics, and WGCNA to connect light‑quality signals to anthocyanin and sugar accumulation. Blue light strongly promoted anthocyanin biosynthesis (notably malvidin‑3‑O‑glucoside and peonidin‑3‑O‑glucoside) and accelerated visible color change while reducing organic acids and paradoxically producing lower soluble sugar levels than red and white treatments. Red light, in contrast, enhanced expression of photosynthesis and carbon fixation genes in berry skin, which the authors propose as the driver of higher sugar accumulation independent of flavonoid pathway activation. WGCNA identified a “dark red” module associated with anthocyanins and a “cyan” module linked to sugars; these implicated VvPIF4 as a negative regulator of anthocyanins under red light (acting downstream of VvPHY via VvCOP1/VvSPA) and that VvHY5, VvMYB90, and VvMYB86 as positive regulators under blue light, forming a photoreceptor‑anchored transcriptional architecture broadly consistent with that of Arabidopsis and apple. Detached clusters in incubators enabled precise spectral control but limit extrapolation to on-vine ripening, particularly for sugar–acid balance, yet the study offers a mechanistic model that connects photoreceptor identity to coordinated changes in pigments, sugars, acids, and volatiles.^31^

Complementing these blue‑ and red‑centric studies, Guan et al. resolved a specific red‑light signaling module in postharvest oriental melon that directly links light perception to ethylene biosynthesis and sucrose accumulation. They show that red light suppresses the transcription factor CmPIF8, which otherwise binds and represses the CmACO1 promoter, thereby relieving inhibition on ACO, increasing ethylene production, accelerating climacteric ripening, and upregulating SPS activity and CmSPS1 expression to elevate sucrose levels. This CmPIF8–CmACO1 axis is supported by qRT‑PCR, enzyme assays, yeast one‑hybrid, GUS, and dual‑luciferase reporter analyses, as well as transient silencing of CmPIF8 in fruit, which phenocopies red‑light treatment in ethylene and sucrose responses. CmPIF8 does not bind the CmSPS1 promoter, indicating that sucrose effects are indirect and ethylene‑mediated and that the presence of three other CmPIFs upregulated by red light introduces a more complex PIF landscape that this study does not fully resolve. Nonetheless, it moves beyond correlation by providing a defined transcriptional node through which red light controls ripening and sugar metabolism in melon under low‑light greenhouse conditions.^32^

Several studies then focus on postharvest responses where spectral quality modulates oxidative metabolism, browning, and senescence. Jin et al. identified a violet LED‑driven MdHY5–MdHYH positive feedback loop that suppresses enzymatic browning in fresh‑cut apple. Violet light (433 nm) activates MdHY5 and MdHYH, which mutually bind and activate each other's promoters and jointly repress MdPPO and MdPOD while activating MdPAL, shifting phenolic metabolism away from oxidative quinone formation towards phenolic accumulation. This regulatory architecture was validated by Y1H, EMSA, ChIP‑qPCR, GUS and dual‑luciferase assays and transient silencing of each gene in apple slices, which abolishes violet‑induced browning suppression, establishing that both factors are necessary for the anti‑browning effect. Metabolomics reveals 390 differentially accumulated metabolites enriched in the phenylpropanoid and flavonoid pathways, which is consistent with the transcriptional pattern, and violet light was the most consistently effective wavelength across the Fuji, Hanfu, and Lvshuai cultivars, suggesting preferential activation of this bZIP module by violet, although the upstream photoreceptor remains unidentified. The dual repressive and activating roles of MdHY5/MdHYH on different targets are empirically documented but structurally unexplained, leaving an open question regarding promoter‑specific cofactor recruitment.^33^

Yang et al. presented a complementary postharvest case in kiwifruit (*Actinidia chinensis “Jinyan”*), where blue light (420 nm) delays softening and preserves ascorbic acid by globally reinforcing ROS‑scavenging networks rather than via a single regulatory node. Blue light reduced MDA, H₂O₂, and O₂⁻; increased AsA and GSH while lowering DHA and GSSG; and upregulated SOD, CAT, and the full AsA–GSH cycle enzymes (APX, MDHAR, DHAR, and GR) over 15 d at room temperature while suppressing AsA oxidase, collectively maintaining membrane integrity and delaying softening. qRT‑PCR of genes drawn from prior transcriptome data confirmed the upregulation of L‑galactose pathway genes for AsA biosynthesis and genes encoding antioxidant enzymes, positioning ROS management as a central mediator of blue‑light‑induced shelf‑life extension. However, unlike the mechanistic depth in the MdHY5/MdHYH apple or CmPIF8–CmACO1 melon studies, no upstream blue‑light photoreceptor or transcription factor has been identified, and the work is best understood as a detailed metabolic characterization rather than a regulatory dissection, especially since it builds on earlier work from the same group showing blue‑mediated suppression of ethylene, starch degradation, and cell wall metabolism in the same cultivar.^34^

Finally, Shi et al. illustrated how spectral light effects can interact synergistically with microbial volatiles to modulate pigment metabolism and delay senescence, using white LED light combined with volatile organic compounds (BVOCs) from *Lysinibacillus fusiformis in* stored broccoli. The combined LED–BVOC treatment maintained a green color (yellowing index 31.3% vs. 100% in dark controls after 5 d), preserved soluble protein and TSS, reduced respiration and lipid peroxidation, and outperformed the individual LED or BVOC treatments. Transcriptomic and metabolomic analyses (LED–BVOCs vs. dark) showed downregulation of chlorophyll degradation genes (BoCLH1/2/3, BoPAO, BoPPH) and corresponding enzymes (Chlase, PPH, MD, Chl‑POX), maintenance of biosynthetic gene expression, reduced biliverdin, and elevated porphobilinogen, indicating coordinated preservation of chlorophyll. In carotenoid pathways, increased BoZEP expression suggested flux redirection toward the lutein cycle and photoprotective zeaxanthin rather than ABA and senescence‑linked derivatives, connecting light energy management with hormone signaling. Flavonoid changes were observed but interpreted mainly as markers of secondary metabolic rebalancing rather than as direct drivers of color. A key limitation is that molecular analyses were performed only on the combined treatment versus dark, so the individual contributions of LED and BVOCs to specific differentially expressed genes and metabolites cannot be disentangled, and the precise modes of action of the identified BVOCs remain uncharacterized.^35^

Taken together, the studies reviewed in this section demonstrate that spectral light quality governs photosynthesis, metabolism, and molecular regulation through tightly coupled but highly context‑dependent mechanisms, yet they also expose important gaps that constrain predictive application and highlight clear priorities for future work. Blue- and violet-enriched spectra emerge as central regulators of secondary metabolism, consistently enhancing carotenoids, anthocyanins, flavonoids, and antioxidant capacity across leafy tissues, fruit surfaces, and whole fruits, but their effects on biomass and primary metabolism range from clearly beneficial to mildly inhibitory, depending on irradiance, spectral background, and temporal delivery pattern, indicating that “more blue” is not a universally optimal prescription and that flux-level validation (rather than transcript or pool sizes alone) is essential for mechanistic interpretation. Red‑light–dominated regimes, often in combination with far‑red, primarily act through PIF‑centered networks to modulate ethylene biosynthesis, the ripening rate, sucrose accumulation, and even ionomic profiles but can simultaneously suppress pigment accumulation or dilute nutraceutical compounds, thereby creating non‑trivial trade‑offs between yield, sweetness, color, and micronutrient content that need to be quantified explicitly rather than treated as secondary effects. Across species and systems, there is a clear convergence on a small set of regulatory hubs, HY5‑like bZIPs, PIFs, ERFs, and ROS/AsA–GSH circuitry, yet most studies still stop at associative multi‑omics or pathway‑level characterization, with only a few providing full causal chains from light quality through defined transcription factors to target genes, metabolites, and measurable quality parameters and even fewer testing whether these mechanisms hold outside model genotypes or highly controlled environments. Methodologically, the literature is constrained by reliance on single cultivars and dwarf lines, monochromatic or overly simplified spectral treatments, short experimental windows, limited replication under production‑like conditions, and incomplete integration of dynamic factors such as diurnal timing, pulse frequency, and interaction with other stimuli (e.g., microbial volatiles), all of which limit cross‑study comparability and make it difficult to separate genuine biological variability from artefacts of experimental design. Addressing these limitations will require longitudinal, multi‑cultivar studies that combine realistic multi‑band LED spectra with systematic variation in intensity and temporal pattern, while embedding mechanistic experiments (photoreceptor mutants or knock‑downs, targeted TF perturbation, flux analyses) into physiologically relevant systems; such work, ideally replicated across contrasting production environments and integrated with economic and energy‑use assessments, will be crucial for translating mechanistic insights into robust, crop‑specific spectral recipes that balance productivity, postharvest performance, and nutritional quality in a quantitatively predictable way.

## Postharvest LED/UV treatments for shelf-life extension and senescence delay

In recent years, postharvest light-based treatments, particularly those utilizing light-emitting diodes (LEDs) and ultraviolet (UV) irradiation, have attracted considerable attention as non-chemical strategies to enhance the shelf-life and quality of fresh produce. These interventions can effectively regulate ripening, delay senescence, reduce microbial decay, and preserve biochemical integrity through wavelength-specific modulation of physiological and metabolic processes. As summarized in Table 3, postharvest LED and UV treatments vary in their effects across horticultural crops, depending on factors such as spectral wavelength, light dose, exposure duration, and crop-specific physiology. Overall, controlled spectral illumination offers a promising complement to conventional cold storage by enabling targeted regulation of ripening, antioxidant metabolism, and stress responses, though its efficacy remains highly dependent on treatment conditions and species-specific responses.

**Table 3.** Effects of postharvest LED and UV light treatments on ripening, senescence, microbial decay, and biochemical composition in horticultural crops. Responses depend on wavelength, dose, exposure conditions, and crop-specific physiology. Symbols denote direction of change (↑ increase; ↓ decrease).

Crop	Light treatment	Key effects	Mechanistic insight	Reference
Tomato (*Solanum lycopersicum* L. *cv. Raf)*	Red (620–625 nm), Blue (460–467 nm) LEDs; UVA (366 nm), UVC (254 nm)	↑ carotenoids, ↑ antioxidant capacity (~30%); UV: no effect	Visible light induces carotenoid biosynthesis; UV is ineffective	[36]
Strawberry (*Fragaria* × *ananassa* Duch.)	405 nm LED	↓ fungal growth, ↑ phenolics, ↑ anthocyanins, ↑ vitamin C	Antimicrobial + metabolic stimulation	[37]
Tomato (*Solanum lycopersicum* L.)	Light + temperature	Accelerated ↑ ripening rate, ↑ sugars, ↑ phenolics, ↑ lycopene	Light response modulated by temperature	[38]
Pakchoi (*Brassica chinensis* L. “Shanghaiqing”)	Red LED	Delayed senescence, ↑ ascorbic acid, ↑ glutathione	Activation of ascorbate metabolism	[39]
Pakchoi (*Brassica campestris* L. ssp. *chinensis*)	White LED (448 nm and 549 nm)	↓ respiration, ↓ MDA, ↑ chlorophyll, ↑ vitamin C	Reduced oxidative damage, maintained antioxidants	[40]
Pakchoi (*Brassica rapa* ssp. *chinensis*), Lettuce (*Lactuca sativa* L.)	Multi-wavelength LEDs	↑ chlorophyll retention, ↑ sensory quality	Synergistic spectral effects	[41]
Banana (*Musa*)	UVC (275 nm)	↓ fungal infection, ↓ black spot	Dose-dependent pathogen inhibition	[42]
Murcott mandarin (*Citrus reticulata Blanco* × *Citrus sinensis Osbeck*)	UVC (275 nm)	>90% ↓ green mold	Dose-dependent antifungal effect	[43]
Kumato® cherry tomatoes (*Solanum lycopersicum*)	Blue–red (465/630 nm), Green–red (525/630 nm), Green–far-red (525/670 nm) LEDs	↑ carotenoids, ↑ phenolics; ↑ weight loss, ↓ firmness	Spectrum-dependent metabolic trade-offs	[44]
Tomato fruits (Panekra)	Red LED, full-spectrum LED	Red: ↑ lycopene, ↑ firmness; Full: ↑ soluble solids	Differential regulation of ripening pathways	[45]
Avocado (*Persea americana*)	Red (660 nm), Blue (450 nm) LEDs	Red: ↓ anthracnose, ↑ antioxidants; Blue: ↑ ripening	Red enhances phenolics; blue accelerates metabolism	[46]

Across multiple climacteric fruit studies, a consistent pattern emerges wherein red and blue LED wavelengths play distinct yet complementary roles in modulating postharvest ripening and carotenoid biosynthesis. Baenas et al. demonstrated that continuous red–blue LED illumination (nine red diodes at 620–625 nm and three blue diodes at 460–467 nm) during refrigerated storage at 6 °C ±1 °C effectively stimulated carotenoid accumulation in Spanish “Raf” tomatoes over seven days. The total carotenoid content increased approximately three-fold in the LED-treated tomatoes compared with the fruits stored in darkness, with E-lycopene showing the most pronounced increase of approximately 5.5-fold and total Z-lycopene isomers increasing nearly three-fold. Notably, UV pre-treatment at 1 kJ/m² using either UVA (366 nm) or UVC (254 nm) produced minimal changes in carotenoid accumulation, and combining UV pre-treatment with LED illumination did not provide significant additional enhancement beyond LED treatment alone, indicating that continuous LED exposure was the dominant factor driving carotenoid biosynthesis during refrigerated storage. While the total phenolic content remained largely unchanged (156–223 mg gallic acid equivalents kg^−1^), both the hydrophilic and lipophilic antioxidant capacities increased significantly by approximately 30%–50% compared with those of the control fruits, which was likely associated with carotenoid accumulation and potentially increased vitamin C levels. Strong positive correlations were identified between lipophilic antioxidant capacity and carotenoid concentrations, particularly lycopene and β-carotene. The color development parameters (a* values, hue angle, ΔE) closely paralleled carotenoid increases, while weight loss, total soluble solids, titratable acidity, and the ripening index showed no significant deterioration, demonstrating that LED exposure maintained overall fruit quality during refrigerated storage.^36^

This wavelength-dependent stimulation of carotenoid biosynthesis under refrigerated conditions aligns with observations by Choi and Park, who investigated the combined effects of temperature and light on tomato (cv. Red 244) ripening under controlled environmental chambers with LED illumination containing blue and red wavelengths in a 2:8 ratio at 400 μmol m^−2^ s^−1^. Their study revealed that tomatoes exposed to high-temperature (30 °C/20 °C day‒night) and light conditions (STL) exhibited the highest lycopene content and ripened most rapidly, whereas fruits stored under low-temperature (20 °C/15 °C) and dark conditions (WTD) showed the slowest ripening and lowest carotenoid accumulation. Importantly, light exposure during ripening promoted uniform red coloration across the fruit surface, whereas tomatoes ripened in darkness developed uneven and mottled coloration, with portions remaining green even at advanced ripening stages, which was attributable to light-regulated carotenoid biosynthesis and phytochrome-mediated pigment metabolism. Soluble sugar accumulation (fructose and glucose) was more strongly affected by light than by temperature, with moderate temperature combined with light (WTL) favoring the highest sugar accumulation. Furthermore, chlorophyll a fluorescence imaging effectively differentiated ripening stages, with parameters such as RFD (relative fluorescence decline), NPQ (non-photochemical quenching), and QY (maximum PSII quantum yield) showing distinct patterns during ripening progression and serving as reliable non-destructive indicators of ripening efficiency and maturity status. The transition from the mature green to the breaker stage was particularly sensitive to environmental conditions, with WTD-treated tomatoes requiring more than three times longer to reach the breaker stage compared with STL-treated fruits, indicating that the environmental regulation of tomato ripening is especially critical during this early transition phase.^38^

Expanding on spectral specificity, Martínez-Zamora et al. systematically compared six lighting conditions, including darkness (control), white LED (20 W m^−2^), blue LED (21.5 W m^−2^, 465 nm), blue plus red (B + R; 11.3 and 10.9 W m^−2^), green plus red (G + R; 11 and 10.2 W m⁻²), and green plus far-red (G + FR; 11 and 11.3 W m^−2^), on Kumato® cherry tomatoes during 13 d of refrigerated storage at 5 °C. Continuous LED illumination increased weight loss and firmness reduction (approximately 30%–35% decrease) compared with darkness, though no chilling injury symptoms were visually observed. Fruits stored under blue and B + R light exhibited enhanced ripening characteristics, with B + R lighting stimulating total carotenoid accumulation by nearly 27%, mainly through increased lycopene (representing nearly 67% of total carotenoids) and β-carotene synthesis. In contrast, the G + R and G + FR treatments enhanced total phenolic content by approximately 30% compared with darkness, with chlorogenic and caffeic acids identified as the predominant phenolic acids and naringenin as the major flavonoid. The total antioxidant compounds (sum of carotenoids, phenolic acids, and flavonoids) were highest under B + R illumination (1142.1 mg kg^−1^ fw), followed closely by blue light treatment, both showing significantly greater antioxidant retention than darkness or white light. These findings complement those of^36^ and^38^ by demonstrating that while blue and red wavelengths synergistically enhance carotenoid biosynthesis, green-spectrum combinations can selectively stimulate phenolic metabolism, suggesting that the wavelength-specific activation of distinct biosynthetic pathways is regulated by different photoreceptor systems, such as cryptochromes (blue light) and phytochromes (red/far-red light).^44^

A parallel set of studies on non-climacteric leafy vegetables reveals somewhat different mechanistic priorities, emphasizing oxidative stress mitigation and chlorophyll preservation rather than ripening regulation. Guo et al. investigated red LED irradiation (6.5 μmol m^−2^ s^−1^, 12-h light/dark cycles) on pakchoi (*Brassica rapa* L. *subsp. chinensis*) stored at 15 °C for 8 d and demonstrated that RL irradiation substantially delayed yellowing and senescence by maintaining significantly higher chlorophyll a, chlorophyll b, and total chlorophyll levels dark-stored controls. The protective mechanism centered on stimulation of the ascorbate‒glutathione (AsA‒GSH) cycle, a major antioxidant defense pathway responsible for maintaining ROS homeostasis. RL-treated pakchoi exhibited markedly lower H₂O₂ and malondialdehyde (MDA) contents, indicating reduced ROS accumulation and lipid peroxidation, while DPPH (2,2-diphenyl-1-picrylhydrazyl) and superoxide anion scavenging activities were significantly higher. Activities of antioxidant enzymes, including superoxide dismutase (SOD), peroxidase (POD), and catalase (CAT), were strongly elevated, and RL treatment significantly increased reduced ascorbic acid (AsA), glutathione (GSH) and the AsA/DHA and GSH/GSSG ratios, reflecting a more reduced cellular redox environment. Critically, RL irradiation enhanced activities and gene expression of AsA-GSH cycle enzymes, including L-galactono-1,4-lactone dehydrogenase (GLDH), ascorbate peroxidase (APX), dehydroascorbate reductase (DHAR), and glutathione reductase (GR). The central role of ascorbic acid biosynthesis was confirmed using lycorine (LYC), an inhibitor of GLDH: when RL was combined with LYC, the protective effects were largely abolished, with pakchoi showing significantly higher H₂O₂ and MDA levels, lower chlorophyll retention, and reduced sensory quality comparable to dark-stored controls, demonstrating that RL-mediated preservation is closely dependent on ascorbic acid biosynthesis and antioxidant cycling.^39^

Zhou et al. similarly examined continuous low-intensity white LED irradiation (10 μmol m^−2^ s^−1^, spectral composition 29% blue, 47% green, 24% red, centered at 448 nm and 549 nm) on pakchoi (*Brassica campestris* L. *ssp. chinensis*) stored at 20 °C for 7 d and observed comparable senescence-delaying effects. The white LED-treated pakchoi maintained greener leaves, firmer stems, lower decay, and extended acceptable shelf-life by approximately 2–3 d, with significantly lower respiration rates throughout storage, indicating delayed metabolic activity. Chlorophyll levels were substantially higher on days 3, 5, and 7 compared with dark-stored controls, and the vitamin C content was better maintained during early storage. Malondialdehyde accumulation was significantly lower, reflecting reduced oxidative stress. Importantly, white LED treatment enhanced activities and gene expression of antioxidant enzymes (POD, CAT, and APX) while simultaneously suppressing chlorophyll-degrading enzymes, including chlorophyllase (Chlase), chlorophyll-degrading peroxidase (Chl-POX), Mg-dechelatase (MD), and pheophytinase (PPH). Gene expression analysis showed reduced expression of the chlorophyll degradation-related genes BrChlase1, BrChlase2, and BrPPH, while expression of the chlorophyll synthesis-associated gene BrHEMA1 was maintained or enhanced. These coordinated regulatory effects, which promote chlorophyll biosynthesis while suppressing catabolism alongside enhanced antioxidant defense, demonstrate that LED irradiation in leafy vegetables operates through dual metabolic regulation distinct from the ripening-associated pathways dominant in climacteric fruits.^40^

Zhu et al. extended this understanding by developing a novel LED-based preservation light source designed according to the chlorophyll absorption spectrum, using spectral superposition of 24 one-watt LEDs of ten different spectral types (blue wavelengths around 420–450 nm and red wavelengths around 640–662 nm) arranged to produce a photosynthetic photon flux density (PPFD) of 35 μmol m^−2^ s^−1^ at 0.92 uniformity. When applied to pakchoi and lettuce stored at 20 °C ± 1 °C for 3 d (12 h daily illumination), the novel light source retained greener leaves and significantly higher a/b color values (correlating with greenness retention) than samples stored under darkness, daylight, or monochromatic red (660 nm) or blue (440 nm) light. The chlorophyll contents were 6.19% and 7.75% higher in pakchoi and 19.67% and 7.57% higher in lettuce compared with darkness and daylight controls, respectively. Compared with monochromatic blue and red light, the novel multi-wavelength source-maintained chlorophyll contents are 15.13% and 5.92% higher in pakchoi and 11.26% and 11.81% higher in lettuce, suggesting that spectral matching with chlorophyll absorption characteristics provides superior preservation compared with single-wavelength approaches. This finding contrasts with the efficacy of monochromatic red LED reported by^39^ and suggests that while monochromatic red light effectively stimulates antioxidant metabolism, multi-wavelength combinations better support chlorophyll biosynthesis and preservation, possibly by simultaneously activating multiple photoreceptor systems and metabolic pathways. The slightly higher water loss observed under LED irradiation across studies by^40^ and,^41^though not always statistically significant, suggests a potential trade-off wherein light-induced stomatal activity or metabolic stimulation may increase transpiration, requiring optimization of light intensity and duration to balance preservation benefits against moisture retention.^41^

Shifting to ultraviolet-based antimicrobial strategies, a distinct preservation mechanism emerges centered on photodynamic inactivation and direct pathogen suppression rather than host metabolic modulation. Chong et al. investigated 405 nm LED illumination (2.68 ± 0.5 mW/cm², continuous exposure) on strawberries inoculated with *Rhizopus stolonifer* or *Botrytis cinerea* and stored at 15 °C for 12 d. The 405 nm LED treatment produced strong antifungal activity, with *R. stolonifer* populations remaining stable at approximately 3.5–4.0 log CFU/g in illuminated strawberries compared with nearly 7.0 log CFU/g in controls after 12 d (approximately 3.4 log reduction), while *B. cinerea* populations showed an approximately 1.9 log reduction. The temperature increase caused by LED exposure was negligible (0.8 °C ± 0.2 °C), confirming that fungal inhibition resulted primarily from photodynamic effects involving endogenous photosensitive compounds such as porphyrins absorbing visible light and generating reactive oxygen species that damage fungal DNA, proteins, and lipids. Importantly, LED treatment did not adversely affect physical quality: mass loss and texture softening were similar between illuminated and control strawberries, and color parameters (a*, b* values) were not significantly altered. However, 405 nm LED illumination positively influenced nutritional properties, with the total phenolic content and Trolox equivalent antioxidant capacity rising more rapidly and to significantly higher levels in illuminated strawberries, particularly after Day 9, while anthocyanin content was significantly higher from Day 6 onward, suggesting LED-stimulated anthocyanin biosynthetic enzyme activity. The vitamin C content declined in illuminated strawberries beginning after Day 9, possibly due to increased utilization of glucose in antioxidant-related metabolic pathways, whereas the control samples did not show significant reductions relative to the initial values. This trade-off between enhanced phenolic/anthocyanin accumulation and vitamin C reduction differs from the antioxidant preservation patterns observed in tomatoes and leafy vegetables under red/blue LED irradiation, potentially reflecting commodity-specific metabolic responses or differences between visible-spectrum and near-UV wavelengths.^37^

In line with these observations, Le et al. developed a UVC LED-based optical irradiation system using 275 nm LEDs arranged in a 4 × 4 matrix array with dome lenses to achieve narrow divergence angles (FWHM reduced from approximately 120° to 22°) and high irradiation uniformity (85.4% simulated, 77.2% experimental) for controlling green mold disease (*Penicillium digitatum*) in murcott fruits stored at approximately 25 °C. Under *in vitro* conditions, UVC doses exceeding 0.30 kJ/m² inhibited more than 90% of *P. digitatum* conidial growth after 72 h, with exposure durations of 1.0, 2.0, 3.0, and 5.0 min resulting in inhibition rates of approximately 94.7%, 93.2%, 91.7%, and 94.7%, respectively, demonstrating substantial suppression at lower UVC doses than those reported in some earlier studies. Under *in vivo* storage conditions, murcotts treated for 5 min (approximately 1.5 kJ/m²) maintained a complete absence of visible disease symptoms throughout seven weeks, while treatments of 3–15 min maintained good fruit quality for approximately four weeks with minimal disease development. Excessively high UVC doses (20-min treatment) did not further improve preservation efficacy and showed some disease development during long-term storage, suggesting that excessive irradiation may increase fruit susceptibility to damage or physiological stress.^43^

Similarly, Le et al. designed a UVC LED cavity (275 nm) for banana preservation to control *Colletotrichum mused-induced anthracnose*, achieving high irradiance uniformity (87.1% for 13 × 13 cm region, 77.0% for 17 × 17 cm region) with a dominant irradiance of approximately 6–9 W/m² in the cavity center. UVC treatment strongly inhibited conidial germination, with inhibition exceeding 90% at doses above 0.36 kJ/m² and reaching approximately 99.3% at 1.08 kJ/m², while mycelial growth inhibition remained more modest (maximum 21.9% at 1.80 kJ/m²), indicating that UVC is particularly effective against early infection stages. However, banana peels were highly sensitive to excessive UVC exposure: the optimal treatment duration was identified as 5 s (approximately 0.030–0.045 kJ/m² for upper-surface treatment), which significantly reduced black spot formation after seven days while maintaining an acceptable peel appearance, whereas prolonged exposure caused undesirable browning and sensory damage. This striking dose sensitivity contrast, in which murcott fruits tolerating 1.5 kJ/m² while bananas require only 0.030–0.045 kJ/m², underscores substantial commodity-specific variation in UVC tolerance and highlights the critical importance of precise dose optimization for each fruit type to balance antimicrobial efficacy against tissue damage.^42^

In a different perspective, Meiramkulova et al. compared red-spectrum LEDs, full-spectrum red-blue-white LEDs, and UV-LEDs for preserving *Panekra tomatoes* during 21 d of storage at 13 °C–15 °C. Red-spectrum and full-spectrum LEDs were applied for 2 h daily, while UV-LEDs were applied for 2 min three times daily. Full-spectrum and UV-LED treatments substantially reduced moisture loss compared with controls, with UV-LED-treated tomatoes demonstrating the lowest overall weight loss and the control samples exhibiting approximately 4.46% weight loss. The LED-treated tomatoes maintained relatively stable dimensions, whereas the control samples showed noticeable shrinkage. Red-spectrum-treated tomatoes retained the highest ascorbic acid concentrations (approximately 1.8 mg/100 mL) compared with approximately 1.0 mg/100 mL in controls, and the full-spectrum LED treatment produced the highest total soluble solids (approximately 7.3 °Brix) compared with approximately 3.9 °Brix in controls. Full-spectrum-treated tomatoes retained approximately 66.2% moisture after 14 d and approximately 59.2% moisture after 21 d, whereas the control samples exhibited considerably lower moisture retention. Although lycopene preservation trends favored LED treatments, particularly red-spectrum LEDs, ANOVA indicated that some differences were not statistically significant, suggesting that preservation effects may be more pronounced for certain quality parameters than others. This study's integration of UV-LED with visible-spectrum LEDs demonstrates that combining antimicrobial UVC action (pathogen suppression) with metabolic stimulation from red/blue wavelengths may offer complementary preservation benefits, though the relative contributions of each wavelength regime require further systematic comparison.^45^

In contrast to visible-spectrum and UVC approaches, red LED light appears particularly effective in stimulating host defense responses against latent fungal infections in climacteric fruits. Mpai and Sivakumar investigated monochromatic red LED (660 nm, 150 μmol m^−2^ s^−1^, 6 h daily) and blue LED (450 nm, 100 μmol m^−2^ s^−1^, 6 h daily) treatments on the avocado cultivars Hass and Fuerte during simulated market storage at 12 °C–14 °C for up to 16 d, comparing these with those of conventional prochloraz fungicide treatment. Red LED irradiation significantly reduced anthracnose incidence in both artificially inoculated and naturally infected fruits, achieving ≤25% incidence in cv. Fuerte and approximately 12% in cv. Hass during prolonged storage, whereas blue LED- and prochloraz-treated fruits showed substantially higher disease levels. Red LED treatment significantly upregulated phenylalanine ammonia lyase (PAL) gene expression, particularly after 12 d, indicating that activation of the phenylpropanoid pathway is responsible for phenolic defense compound biosynthesis. Concurrently, red LED-treated fruits retained significantly higher epicatechin levels, an important antifungal phenolic compound known to suppress latent Colletotrichum infections. Red LED irradiation also significantly suppressed lipoxygenase (LOX) gene expression associated with membrane degradation, lipid peroxidation, and ripening-related senescence, contributing to delayed ripening and improved membrane stability. Approximately 70%–80% of red LED-treated fruits remained firm after 8–12 d compared with accelerated ripening occurred under blue LED exposure, which was associated with a reduced D-mannoheptulose content, a C7 sugar that inhibits glycolysis and delays ripening. Red LED-treated fruits maintained significantly higher D-mannoheptulose concentrations, particularly after 8–12 d, thereby contributing to prolonged shelf-life. Additionally, red LED irradiation significantly enhanced ascorbic acid accumulation and antioxidant activity and increased the oleic acid content relative to fungicide-treated fruit. This comprehensive defense activation by red LED, involving upregulated PAL expression, enhanced phenolic retention, suppressed LOX activity, maintained ripening-inhibitory sugars, and improved the antioxidant status, which demonstrates a multifaceted host-mediated resistance mechanism distinct from the direct pathogen inactivation achieved by UVC irradiation and suggests that red LED exposure could potentially replace conventional fungicide applications for controlling postharvest diseases in certain climacteric fruits.^46^

Collectively, these studies demonstrate that LED-based postharvest preservation efficacy depends critically on wavelength selection, light intensity, exposure duration, and commodity-specific physiology. Red and blue wavelengths synergistically enhance carotenoid biosynthesis and antioxidant accumulation in climacteric fruits,^36^ ^,^ ^38^ ^,^ ^44^ while red LED specifically stimulates ascorbate‒glutathione cycling in leafy vegetables^39^ and activate host defense responses against fungal pathogens in avocados.^46^ Multi-wavelength combinations matching chlorophyll absorption spectra provide superior preservation compared with monochromatic treatments,^41^ whereas UVC irradiation operates through direct pathogen inactivation requiring precise dose optimization to prevent tissue damage, ranging from 0.030–0.045 kJ/m² for bananas to 1.5 kJ/m² for citrus.^42^ ^,^ ^43^ Light intensity requirements vary substantially, with low-intensity illumination (6.5–35 μmol m^−2^ s^−1^) effective for leafy vegetables and higher intensities (100–400 μmol m^−2^ s^−1^) needed for fruit ripening modulation. However, several limitations constrain commercial implementation: potential trade-offs, including increased water loss and vitamin C decline under certain treatments;^37^ ^,^ ^40^ ^,^ ^44^ a lack of systematic wavelength comparisons within unified experimental frameworks; a limited evaluation of long-term storage beyond 2–4 weeks, insufficient attention to economic feasibility and scalability, and inadequate characterization of the molecular mechanisms linking photoreceptors to metabolic responses. Future research should prioritize controlled comparative studies across multiple wavelengths and commodities, extended storage trials, techno-economic analyses, consumer acceptance testing, and detailed mechanistic investigations to transition LED preservation technologies from laboratory demonstrations to widespread commercial applications.

## Enhancement of bioactive compounds, antioxidants, and nutritional quality

Light quality plays a pivotal role in regulating the biosynthesis, accumulation, and preservation of bioactive compounds in fruits and vegetables. Modern LED technology enables precise spectral control with high energy efficiency and minimal environmental impact, positioning it as a valuable tool for both cultivation and postharvest management. As summarized in Table 4, different LED light treatments significantly influence the bioactive compound content, antioxidant capacity, and overall nutritional quality across various horticultural crops. These effects are strongly dependent on wavelength, species, and experimental conditions, reflecting complex light–plant interactions that shape metabolic outcomes. Importantly, evidence from previous studies indicates that light exposure does not uniformly enhance nutritional attributes but rather modulates metabolic pathways in a wavelength-specific manner, thereby underscoring the need for context-aware optimization of spectral treatments.

**Table 4.** Summary of the effects of LED light treatments on bioactive compounds, antioxidant capacity, and nutritional attributes in fruit and vegetable crops, with associated mechanistic interpretations. Responses are dependent on spectral quality, species, and experimental context. Symbols: ↑ denotes an increase or stimulation; ↓ denotes a decrease or inhibition of the reported parameter.

Crop	Light treatment	Key effects	Mechanistic insight	Reference
Phlegrean mandarin (*Citrus reticulata* Blanco)	Red–blue LED (red: 630 nm, blue: 460 nm; 60 R:40B); White light	Red–blue: altered ascorbic acid, flavonoids, anthocyanins, carotenoids; White: ↑ specific polyphenols	Light quality redistributes metabolic flux among compound classes rather than increasing total antioxidants	[47]
Tomato (*Solanum lycopersicum* L. *cv. Microtom*)	Full-spectrum vs Red–blue (60 R:40B)	Full-spectrum: ↑ polyphenols, flavonoids, tannins, ascorbic acid, lycopene; Red–blue: ↑ carotenoids, antioxidant capacity	Broad spectrum supports metabolic balance; red–blue promotes pathway-specific responses	[48]
Raspberry (*Rubus idaeus* L.) and Blackberry (*Rubus fruticosus* L.)	Intermittent LED pulses of 15 min every 2 h (green: 525 nm, blue: 470 nm, red: 625 nm)	Raspberry: ↑ organic acids, ascorbic acid; ↓ breakdown (stronger under green/blue); Blackberry: limited response	Species-dependent responses linked to metabolic differences	[49]
Tomato (*Solanum lycopersicum*) (preharvest)	Red–blue (640/455 nm; 1:1)	↑ yield, lycopene, phenolics, vitamin C, soluble solids	Light regulates developmental programming and metabolite accumulation	[50]
Fresh-cut amaranth (*Amaranthus dubius* L.*)*	Blue LED (460 nm)	↑ chlorophyll, ascorbic acid; ↓ microbial growth; extended shelf-life	Activation of AsA–GSH antioxidant cycle	[51]
Table grapes (*Vitis vinifera cv. Kyoho*)	Red LED (625 nm)	↓ weight loss, browning, lipid peroxidation, ROS; ↑ phenolics, antioxidant enzymes	Reduced oxidative stress and enhanced antioxidant defense	[52]
Japanese eggplant (*Solanum melongena* L.)	Red–blue LED for 48 h (red: 650–660 nm; blue: 450–460 nm; 70 R:30B)	Peel: ↑ phenolics, flavonoids; Flesh: ↑ carotenoids	Tissue-specific metabolic responses	[53]
Bell pepper (*Capsicum annuum* L.*)*	Blue–red LEDs (450 nm, 660 nm) ± UV-B pre-treatment	↑ carotenoids, antioxidant capacity (enhanced with UV-B)	Interaction of light and UV-B stress enhances antioxidant pathways	[54]
Hot pepper (*Capsicum annuum* L.)	Red (660 nm); far-red (730 nm)	Red: ↑ carotenoids; Far-red: ↑ capsaicin	Phytochrome-mediated responses regulate carotenoid and capsaicin pathways	[55]

The body of evidence on postharvest light treatments consistently indicates that light quality, intensity, timing, and commodity-specific physiology interact to modulate bioactive compounds, antioxidant status, and nutritional quality, but the direction and magnitude of these effects are clearly context-dependent rather than universally predictable across crops. In *Phlegrean mandarin*, Costanzo et al. showed that continuous postharvest LED illumination at 150 ± 20 µmol photons/m^−2^ s^−1^ for seven days at 20 °C ± 2 °C under a red–blue (RB, 60:40) regime substantially enhanced a broad suite of antioxidants in both the peel and pulp relative to white (W) light and dark control fruit, including increases in total polyphenols (peel), flavonoids (approximately 32% in the pulp), anthocyanins (approximately 81% in the pulp), carotenoids (approximately 36% in the pulp and 32% in the peel), ascorbic acid, and total antioxidant capacity, as assessed by FRAP. This effect was mechanistically linked to RB-induced activation of key biosynthetic enzymes such as PAL, CHS, DFR, and PSY, suggesting that dual red/blue stimulation can broadly upregulate the phenylpropanoid and isoprenoid pathways in citrus peel and pulp tissues. At the same time, the same study highlighted that W light, despite lower overall antioxidant indices, selectively promoted specific high-value polyphenols (chlorogenic acid, quercetin, rutin, naringin, valoneic acid, hesperidin), which the authors associated with its lower blue fraction (21%) and possibly reduced repression of miR393/394/395-regulated rutin and quercetin biosynthesis compared with the higher blue proportion (40%) under RB. The divergent responses of FRAP and DPPH in mandarin, with higher DPPH inhibition in control and W fruits than in RB-treated fruits, underscore that different antioxidant assays capture distinct redox mechanisms and can be differentially influenced by the structural diversity of polyphenols, a methodological nuance that recurs across several studies in this literature.^47^

A similar view of light quality as a metabolic “lever” rather than a simple on–off switch for antioxidant enrichment emerges from work on tomato and other solanaceous crops, although the specific spectral optima differ. In the dwarf tomato cultivar “Microtom”, Hay Mele et al. exposed plants from sowing to fruit ripening under three lighting regimes, white fluorescent (FL), full-spectrum (FS), and RB LEDs, and quantified an extensive panel of bioactive markers, including carotenoids, lycopene, polyphenols, flavonoids, condensed tannins, anthocyanins, ascorbic acid, ferric reducing antioxidant power (FRAP) and DPPH antioxidant activities, soluble proteins, carbohydrates, and LC-MS/MS-resolved polyphenols. In contrast to the mandarin study, where RB was broadly superior, FS in “Microtom” produced the highest accumulation of polyphenols, flavonoids (a 666.2% increase relative to RB), condensed tannins, ascorbic acid, and lycopene, while RB maximized total carotenoids and FRAP, and FL preserved anthocyanins, soluble proteins, and carbohydrates. Spectral correlation analysis indicated that the blue (400–470 nm) and orange–red (610–670 nm) bands were positively correlated with carotenoid accumulation, whereas the green (500–550 nm) and far-red (710–760 nm) bands were more strongly associated with polyphenol and flavonoid enrichment, and blue light itself correlated negatively with lycopene, suggesting that cryptochrome-linked signaling may suppress some branches of carotenoid biosynthesis under high blue flux. Mechanistically, the authors invoked activation of phytochrome and cryptochrome signaling (including CRY1a) and the upregulation of PAL, CHS, CHI, and FLS in the phenylpropanoid pathway, although these hypotheses remain to be substantiated by direct expression or enzyme assays.^48^ Notably, the discordance between the FRAP and DPPH responses in tomato, which the authors rationalized based on proton-donating versus electron-transfer antioxidant mechanisms, mirrors the assay-dependent patterns observed by^47^ in mandarin, reinforcing that conclusions about “total antioxidant capacity” are sensitive to the choice of metric.

The importance of crop- and even cultivar-specific responses is further emphasized by short-duration, pulsed LED interventions in small fruits. Ganganelli et al. evaluated 15-min pulses every two hours of green (525 nm), blue (470 nm), red (625 nm), and red + blue combinations on raspberries (“*Tulameen”*) and blackberries (“Brazos”) during 14 d of storage at 4 °C, explicitly selecting a pulsed regime to mitigate the potential negative effects of continuous light on water loss and browning. In raspberries, the blue and red + blue treatments markedly reduced internal breakdown at day 14 (≈4%–5% vs ≈ 24% in the controls) and, together with green light, increased quinic and malic acids at day 7 and citric acid at day 14, while green and blue also elevated ascorbic acid at day 7, which is consistent with light-stimulated ascorbate biosynthesis via the myo-inositol or D-galacturonate pathways. However, respiration rates remained higher in LED-treated raspberries than in controls, an observation the authors interpreted as sustained metabolic activity and delayed senescence but which also admits alternative interpretations, given that elevated respiration can reflect stress responses. In contrast, in blackberry, most measured changes in ascorbic and organic acids were attributable to cold storage alone, with only minor LED effects and no consistent reduction in internal breakdown beyond a modest red-light effect at day 14, leading the authors to explicitly caution against extrapolating efficacy from one Rubus species to another. These findings complement the species-specific contrasts seen between citrus and tomato and highlight that even when the same wavelengths are applied in conceptually similar pulsed or continuous regimes, the resulting nutritional and physiological outcomes can be markedly different across fruit types.^49^

The timing of light application relative to the developmental stage can also alter both the direction and magnitude of responses in carotenoid-rich fruits. Ngcobo et al. investigated 48-h red (638 nm) and blue (454 nm) LED treatments at 118 µmol m^−2^ s^−1^ applied to two cherry tomato cultivars (“*Cherry Little Wonder*” and “*Goldilocks”*) harvested at the mature-green or turning stages, followed by 21 d of storage at room temperature. Red light was more effective than blue light in enhancing the a* coordinate (redness) in the red cultivar, while both wavelengths increased b* (yellowness) in the yellow cultivar, with the strongest effects consistently observed when the fruit were treated at the mature-green stage, when chloroplast-to-chromoplast transitions are still underway. The authors linked red light-driven lycopene accumulation to phytochrome-mediated suppression of PIFs and subsequent upregulation of PSY1 and proposed that blue light-induced β-carotene accumulation may reflect cryptochrome-mediated overexpression of CRY1a and stimulation of lycopene-β-cyclase (LCYB), although direct postharvest evidence for blue effects on lycopene remained sparse prior to this work. Both LEDs increased the total soluble solids at both maturity stages without significantly affecting firmness or mass loss, whereas chlorophyll degradation was accelerated by both wavelengths only in mature-green fruit, where the baseline chlorophyll levels were high. Together with the mandarin and “Microtom” studies, these results suggest that, beyond spectral composition, the developmental window during which fruit are exposed to light strongly conditions, whether treatments primarily enhance pigment biosynthesis, accelerate chlorophyll breakdown, or shift sugar metabolism, and that these windows differ across species and cultivar backgrounds.^50^

In leafy vegetables, light intensity appears as a key determinant of the balance between oxidative defense enhancement and potential side effects such as water loss. Jin et al. analyzed freshly cut amaranth subjected to continuous blue LED irradiation (460 nm) at 10, 20, and 30 µmol/(m² s) (T1, T2, T3) versus dark controls during 12 d at 4 °C, coupling biochemical and microbiological endpoints, including 16S rDNA profiling. They reported a clear intensity-dependent hierarchy (T3 > T2 > T1 > control) across sensory scores, chlorophyll retention, soluble solids; ascorbic acid, and the activities of antioxidant enzymes such as APX, GR, SOD, and POD, with T3 extending shelf-life by 2–3 d relative to that of the controls and markedly lowering malondialdehyde accumulation, which is consistent with more effective suppression of oxidative membrane damage. The mechanistic interpretation emphasized the activation of the AsA–GSH cycle, with positive correlations between ascorbate content and APX/GR activities, supporting the view that blue light promoted the recycling of oxidized ascorbate rather than solely de novo synthesis. Concomitantly, microbial counts were significantly reduced under T3, and 16S analyses identified Pseudomonas as the dominant spoilage genus, reaching 56.93% relative abundance in the controls by day 12, with blue light-mediated ROS generation via endogenous photosensitizers proposed as a plausible bactericidal mechanism. Yet, the same intensity gradient also drove an increase in weight loss (T3 > T2 > T1 > control), attributed to blue light-induced stomatal opening and transpiration, illustrating that antimicrobial and antioxidant benefits must be weighed against dehydration risks when defining commercially acceptable intensity thresholds, particularly for minimally processed leafy products.^51^

In berries and other non-climacteric fruits, red light treatments can act both as senescence modulators and as inducers of secondary metabolites, though again within a narrow temporal window. Nassarawa et al. applied continuous red LED irradiation (625 nm, 454 LUX) to table grapes (cv. Kyoho) stored at 22.5 °C ± 2.5 °C for six days and found that treated fruit retained greater firmness (2.67 N higher than that of controls at day 6), higher L* values, reduced weight loss, and less rachis chlorophyll degradation, with the latter being particularly relevant for consumer acceptance. Red light reduced the levels of oxidative stress markers such as malondialdehyde, hydrogen peroxide, and superoxide anions relative to controls, and enhanced activities of POD, CAT, and APX, which is consistent with a bolstered ROS-scavenging capacity; the authors hypothesized that phytochrome-mediated antagonism of ethylene-associated senescence pathways may underlie these effects, although ethylene was not directly measured. On the compositional side, red light markedly increased the content of anthocyanins (from 31.71 to 79.64 mg g^−1^ over six days) and elevated the total phenolics (36.80 mg g^−1^) and flavonoids (20.56 mg g^−1^) compared with those of the controls, which was reflected in higher DPPH and FRAP values. UPLC-DAD-MS profiling identified increased levels of rutin, quercetin, epicatechin, proanthocyanidin B1, gallic, caffeic, p-coumaric, and 3,4-dihydroxybenzoic acids under red light, adding compound-level detail to the aggregate phenolic increases. However, the study used a single cultivar, a six-day ambient storage period, and did not compare multiple wavelengths, so although it supports the potential of red LEDs to simultaneously mitigate oxidative damage and stimulate phenylpropanoid-derived bioactives in table grapes, it does not by itself establish the relative advantage of red over alternative spectra or over refrigerated storage conditions.^52^

Work on Japanese eggplant further illustrates that combined spectra may preferentially enhance specific phenolic subclasses and that the tissue compartment strongly influences outcomes. Jarerat et al. exposed “Shikon Sendai Naga” eggplants to 48 h of red (650–660 nm), blue (450–460 nm), or red + blue (70:30) LEDs and separately analyzed peel and flesh, focusing on phenolics, flavonoids, chlorogenic and gallic acids, anthocyanins, carotenoids, antioxidant activity (DPPH, FRAP), and basic quality traits. The red + blue regime most strongly elevated total phenolics and flavonoids in the peel (821.86 mg GAE/100 g FW and 595.98 mg CE/100 g FW, respectively) and drove the largest increases in chlorogenic acid in both the peel and flesh (35.02 and 58.59 mg/100 g FW, 3.7- and 2.3-fold above controls) and gallic acid in the peel (14.25 mg/100 g FW), with corresponding increases in the DPPH and FRAP, suggesting a positive relationship between phenolic enrichment and antioxidant capacity. The authors attributed these effects to complementary activation of phytochrome (via red) and cryptochrome/phototropin (via blue) signaling, converging on phenylpropanoid pathway activation, though no mechanistic data were collected. Interestingly, anthocyanin accumulation in the peel responded the most to monochromatic red or blue, while carotenoids in the flesh were more effectively enhanced by red alone, implying that the synergistic benefits of combined red + blue loops are not uniform across all pigment classes. The short, 48-h experimental window and the absence of extended storage tracking mean that it remains unclear whether these phenolic gains persist over time and whether they translate into sensory or textural changes, but the study supports the idea that combined spectra can be used to target specific phenolic profiles in eggplant, particularly in peel-rich functional food preparations.^53^

Two pepper studies illustrate how combining UV and LED photoperiods or explicitly manipulating phytochrome status can redirect carotenoid and capsaicinoid biosynthesis in harvested Capsicum fruit. In red bell pepper “Angus”, Martínez-Zamora et al. combined a UV-B pre-treatment (9 kJ m^−2^) after six days of cold storage at 7 °C with three retail photoperiods over four days at 20 °C: fluorescent plus darkness (FL + D), continuous fluorescent (FL + FL), or fluorescent plus blue–red LEDs (FL + BR LED), thereby simulating commercial conditions from transport to supermarket display. FL + BR LED consistently outperformed the other photoperiods in stimulating β-carotene isomer accumulation (13-cis-, all-trans-, 9-cis-β-carotene) and, when preceded by UV-B, further enhanced capsanthin and its esters (including capsanthin laurate) by roughly 22%–38% immediately after UV-B and an additional ≈18% under FL + BR LEDs, with capsanthin derivatives representing around 64.5% of the total carotenoids. The proposed mechanism involves UVR8 activation by UV-B and cryptochrome and phytochrome responses to blue and red light converging on the COP1/HY5 axis and upregulating PSY, thereby boosting carotenoid flux, with partially additive or synergistic effects depending on the carotenoid subclass. Importantly, these nutraceutical gains were achieved without compromising weight loss, firmness, color, or sensory acceptability, and the authors estimated that replacing a full-night fluorescent regime with BR LEDs could cut lighting costs by about 71%, indicating potential economic as well as nutritional benefits.^54^

Taking a more mechanistic approach, Pashkovskiy et al. used green pepper fruit with still-functional chloroplasts and photosystems to investigate how red (RL, 660 nm) and far-red (FRL, 730 nm) light and low temperature (4 °C vs 25 °C dark) shape the Pfr:Pr ratio of phytochrome and, in turn, carotenoid and capsaicinoid biosynthesis via the regulation of PAL, CAM, ACS, FATa, KAS, CAS, and related genes. The experiments showed that RL for 24 h strongly induced carotenoid accumulation (3.6-fold increases in total carotenoids and xanthophylls, especially zeaxanthin, epoxy-lutein, and cis-mutatoxanthin, with a relative decline in β-carotene), which the authors interpreted as a photoprotective response to RL-accelerated photosynthesis and associated oxidative stress rather than purely nutritional enhancement. In contrast, FRL and low temperature, both of which shift phytochrome into the inactive Pr form, induced more than eight-fold increases in capsaicin after 72 h and robust upregulation of PAL, KAS, and FATa, suggesting that phyB acts as a dual light and temperature sensor that modulates capsaicinoid biosynthesis. Measurements of photochemical efficiency (Fv/Fm, Y(II)) indicated that sustained RL and RL + FRL treatments reduced photosystem performance, implying that while RL can be used to boost carotenoids, it may also accelerate senescence and assimilate depletion, whereas FRL or cold primarily boost capsaicinoids with different impacts on photosynthetic status.^55^ Although the study was conducted in a single cultivar under controlled conditions and did not track sensory quality, it provides a mechanistic framework that is conceptually consistent with the more applied findings of^54^ namely, that specific spectral regimes can be tuned to preferentially enrich either carotenoids or capsaicinoids in pepper fruit, potentially even at the consumer level (e.g., via in-fridge LEDs), if issues around energy cost and product quality are addressed.

Taken together, these studies indicate that postharvest light-based strategies can meaningfully enhance or reconfigure bioactive compound profiles, antioxidant status, and related nutritional attributes, but they also reveal important limitations and unresolved questions that temper broad generalization. Many experiments have been conducted on single cultivars under controlled conditions, often with relatively short storage durations and modest replicate numbers, which constrains extrapolation across genotypes, production environments, and commercial supply chains. In addition, the diversity of spectral qualities (from monochromatic blue or red to full-spectrum and combined RB regimes), intensities, and exposure patterns (continuous versus pulsed, hours versus days) makes direct cross-study comparisons difficult and suggests that current evidence is still fragmented, with crop-specific “optima” defined empirically rather than grounded in unified mechanistic models. Several studies also report trade-offs, such as improved antioxidant enzyme activity and microbial suppression at the cost of increased water loss in cut amaranth or sustained respiration in LED-treated raspberries, that may reflect either delayed senescence or stress, highlighting that gains in biochemical markers do not automatically translate into net quality improvements and must be evaluated alongside texture, appearance, and weight loss outcomes. Assay-related constraints further complicate interpretation: discrepancies between FRAP and DPPH within the same sample sets underscore that “antioxidant capacity” is an assay-dependent construct, while a reliance on bulk measures of phenolics or carotenoids, without always resolving individual compounds or their bioavailability, limits nutritional inference. Mechanistically, although several studies propose plausible pathways involving PAL, CHS, PSY, APX, GR, phytochromes, cryptochromes, UVR8, and COP1/HY5, direct evidence linking light treatments to specific gene expression changes, enzyme activities, and, ultimately, to consistent phenotypic traits remains incomplete in many cases, and protein-level and post-translational regulation are rarely addressed in the postharvest context. Looking forward, a key research priority will be to integrate molecular, biochemical, and whole-fruit or vegetable physiology into multi-factorial experimental designs that systematically vary spectrum, intensity, timing, and temperature across multiple cultivars and realistic logistics scenarios (e.g., transport, storage, and retail), while also incorporating techno-economic and life-cycle assessments to evaluate scalability and energy costs. Such studies would support the development of crop- and product-specific light “recipes” that not only maximize targeted bioactive compounds and antioxidant defenses but also maintain or improve sensory quality, minimize losses, and remain economically viable and operationally compatible with existing postharvest systems, thereby addressing the reviewer's call for deeper mechanistic integration and clearer practical relevance within this section.

## Light-mediated control of fruit ripening, softening, and energy metabolism

Light-emitting diode (LED) technologies have recently gained attention as effective non-chemical strategies for regulating postharvest physiology, ripening kinetics, and quality maintenance in horticultural produce, with wavelength-dependent treatments eliciting diverse crop-specific responses in respiration, ethylene production, pigment accumulation, antioxidant capacity, and energy metabolism across climacteric and non-climacteric fruits. As outlined in Table 5, wavelength-dependent LED treatments elicit diverse crop-specific responses, including alterations in the respiration rate, ethylene evolution, pigment accumulation, antioxidant capacity, and energy metabolism.

**Table 5.** Effects of LED light treatments on postharvest physiology and metabolism in horticultural crops. Crop-specific responses and underlying mechanisms are summarized. Arrows indicate direction of change (↑ increase; ↓ decrease).

Crop	Light treatment	Key effects	Mechanistic insight	Reference
Avocado (*Persea americana)*	UV/near-visible LEDs (365, 385, 395, and 405 nm)	Fluorescence correlates with ripening	Reflects compositional changes; corrected for surface variability	[56]
Cherry tomatoes (*Solanum lycopersicum var. cerasiforme*)	Broad-spectrum (400–700 nm)	Delayed luminescence tracks ripening; distinguishes postharvest vs field ripening	Indicates differences in metabolic organization	[57]
Strawberries (*Fragaria *×* ananassa*)	Blue LED (446 nm)	↓ respiration, ↓ ethylene, delayed senescence; maintained firmness and metabolites	Enhanced antioxidant enzymes; reduced ROS and cell wall degradation	[58]
Purple-fruited pepper (*Capsicum annuum*)	Blue light (400–500 nm)	↑ anthocyanins; ↓ ethylene pathways	Pigment accumulation is partially uncoupled from ripening	[59]
Brazil bananas (*Musa* spp.)	Red LED (655 nm)	↓ chlorophyll, ↑ carotenoids, and color development	Enhanced respiratory and energy metabolism enzymes	[60]
Table grapes (*Vitis vinifera* L.)	Red (660 nm) vs Blue (450 nm) LEDs	Red: ↑ sugars, phenolics; Blue: ↑ sucrose cleavage; Both: ↑ TSS, acidity, GABA	Light-specific modulation of sugar and GABA metabolism	[61]
Table grapes (*Vitis vinifera*)	Red (630 nm), Blue (450 nm), Green (530 nm) LEDs	Red: ↑ anthocyanins; All: ↓ chlorophyll degradation	Reduced ATP-related metabolites suggest metabolic suppression	[62]
Strawberries (*Fragaria* × *ananassa*)	Blue (460 nm), Red (660 nm), Red–Blue (3:1)	↑ growth, yield, anthocyanins	Strong cultivar-dependent responses	[63]
Fresh-cut pakchoi (*Brassica rapa*)	Red (660 nm), Violet (405 nm) LEDs	Maintained chlorophyll and ascorbic acid; ↑ antioxidant activity	Linked to antioxidant enhancement and microbial modulation	[64]
Strawberry (*Fragaria* x *ananassa*)	Blue light	No effect on germination; altered stomata	Indicates localized structural response	[65]

Rather than acting through a single conserved pathway, the available evidence indicates that LEDs modulate interconnected metabolic and signaling networks, and comparisons among studies highlight that the light spectrum, intensity, exposure duration, cultivar, and storage conditions together determine whether ripening is accelerated, delayed, or qualitatively modified. Roosta et al. investigated the influence of supplemental LED spectra on growth performance, nutrient uptake, and fruit quality in four hydroponically grown strawberry cultivars (“*Sabrina”, “Albion”, “Parous”,* and *“Camarosa”*), comparing monochromatic blue (460 nm), monochromatic red (660 nm), and combined red/blue (3:1) supplementation to ambient light at a photosynthetic photon flux density of approximately 215 ± 5 μmol m^−2^ s^−1^ under an 11 h/13 h light–dark photoperiod. The authors reported pronounced cultivar-specific responses: “*Parous”* and *“Camarosa*” showed increased fresh and dry biomass of leaves, roots, and crowns under LEDs, while “Albion” in particular exhibited higher fruit number, length, mass, and early yield under combined red/blue light, accompanied by enhanced photosynthetic efficiency, stomatal conductance, chlorophyll accumulation, and CO₂ assimilation, and improved fruit quality traits such as total soluble solids and anthocyanin content, with blue light especially stimulating anthocyanin accumulation in “*Camarosa”*. Nutrient analysis further revealed elevated potassium, magnesium, and iron accumulation under spectral supplementation, leading the authors to conclude that combined red/blue LEDs can improve biomass, photosynthetic performance, early fruit production, mineral nutrition, and nutritional quality in greenhouse strawberries, albeit with large genotype-dependent variation in the magnitude of response.^63^

Complementing this whole-plant study, Pang et al. focused on *in vitro* systems and established a micropropagation protocol for Cameron Highlands white strawberry (*Fragaria *×* ananassa*) seedlings under different LED spectra (*cool white, red, blue*, and c*ombined red + blue*) and plant growth regulator regimes, thereby linking light quality during early developmental stages to subsequent plant performance. In their work, mature red achenes sterilized with 30%–40% Clorox showed similar germination rates (78.77%), but seedling morphology differed: red and blue LEDs induced elongated stems with relatively weak root and leaf development, whereas white and especially red + blue LEDs, promoted more balanced growth with healthier shoots, roots, and earlier attainment of the five-leaf stage. SEM analyses indicated that overall, leaf tissue organization was not adversely affected by the tested light spectra, though blue light caused uneven and slightly damaged stomatal structures; subsequent shoot multiplication on full-strength Murashige and Skoog medium showed optimal shoot induction and elongation at 8 μM thidiazuron (67.5% shoot induction, 6.50 ± 1.05 shoots per explant), while 1 μM indole-3-butyric acid maximized rooting without excessive callus. Pang et al. demonstrated that combining optimized LED spectra (particularly red + blue) with appropriate cytokinin and auxin levels enables efficient, large-scale micropropagation of white strawberry, with successful acclimatization and 100% survival in a hydroponic system and fruiting within four months after transfer. Although conducted under *in vitro* rather than postharvest conditions, these findings align with^63^ by indicating that red/blue combinations generally favor balanced shoot and root development, which could indirectly influence later fruit ripening and quality through their effects on plant vigor and morphogenesis.^65^

Postharvest responses to light spectra have been examined in detail in purple-fruited *Capsicum annuum* (accession #3124) by Liu et al., who investigated how white-red LED intensity (0, 80, 160, 320 μmol m⁻² s⁻¹) and the blue light fraction (24%–99% at 80 μmol m^−2^ s^−1^) influence anthocyanin accumulation and ripening during 28 d of storage. They found that increasing the total white‒red light intensity had a limited impact on the anthocyanin content, whereas increasing the blue light fraction strongly enhanced anthocyanin retention and delayed ripening, with 72% blue light inducing the highest anthocyanin synthesis rate and 99% blue maintaining the highest anthocyanin content at the end of storage. Kinetic modeling and transcriptome data indicated that blue light mainly stimulated anthocyanin biosynthesis rather than reducing degradation, which was supported by the upregulation of anthocyanin biosynthetic genes (CaMYB, CaCHS, CaDFR, CaANS, and CaUFGT) and the enrichment of phenylpropanoid and flavonoid pathways under 72% blue, while ripening- and senescence-related genes (NCED1, NCED2, NOR, and RIN) were downregulated. In parallel, soluble sugar accumulation and chlorophyll degradation progressed more slowly under higher blue fractions, which was consistent with delayed ripening, whereas capsanthin accumulation was only weakly influenced by light treatment. On this basis, Liu et al. proposed that blue-enriched light promotes anthocyanin biosynthesis but slows general ripening, suggesting that such regimes may be suitable when prolonged anthocyanin retention and delayed senescence are desired but may be less appropriate when full ripening of anthocyanin-rich peppers is the primary objective.^59^

Two studies by Nassarawa and co-workers further extended the understanding of how postharvest LEDs modulate primary and secondary metabolism, as well as energy metabolism, in grape berries (*Vitis vinifera* L. *cv. Kyoho*) during cold storage. Nassarawa and Luo exposed grape clusters to red (660 nm) or blue (450 nm) LEDs (500 lx, 4 °C, 21 d) and evaluated sugar metabolism, phenolic biosynthesis, and γ-aminobutyric acid (GABA) metabolism relative to dark-stored controls, thereby dissecting how different spectra influence carbohydrate and amino acid dynamics alongside phenolic accumulation. Both red and blue LEDs increased the total soluble solids while slowing the decline in titratable acidity, but red light produced higher total phenolic, flavonoid, and anthocyanin contents at the end of storage; these effects coincided with elevated activities of phenylpropanoid pathway enzymes (PAL, TAL, C4H, and 4CL), suggesting an overall enhancement of phenolic biosynthesis under red light. Red light also promoted higher glucose, fructose, and sucrose concentrations through the modulation of sucrose synthase (cleavage) and acid invertase, whereas blue light preferentially enhanced sucrose synthesis-related activity; both treatments increased the GABA content via elevated glutamate decarboxylase activity and decreased GABA-transaminase, with blue light inducing the highest GABA levels (36.94 µmol g⁻¹) by the end of storage.^61^ In a complementary study, Nassarawa et al. subjected 612 Kyoho grape clusters to continuous red (630 nm, 13 W), blue (450 nm, 6 W), green (530 nm, 11 W) LEDs or darkness (0 °C, 28 d) and monitored their color, anthocyanin and phenolic contents, respiration, chlorophyll degradation, anthocyanin biosynthesis-related enzymes, ATP-related energy metabolism, respiratory enzymes, and NAD/NADP levels. Red light again emerged as the most effective treatment for maintaining visual quality and color indices, preserving higher anthocyanin (0.99 g kg^−1^) and phenolic contents (4.20 g kg^−1^) than blue, green, or control treatments, and reducing the activities of chlorophyll-degrading enzymes (chlorophyllase, Mg-dechelatase, pheophytinase, and chlorophyll-degraded peroxidase), thereby delaying chlorophyll loss. Importantly, red LED irradiation maintained higher ATP and ADP levels and energy charge and sustained elevated activities of energy metabolism enzymes such as H⁺-ATPase, Ca²⁺-ATPase, cytochrome oxidase, and succinate dehydrogenase, suggesting improved cellular energy status and respiratory function during storage. Together, these two studies indicate that red light can simultaneously stimulate phenylpropanoid metabolism, sustain sugar and energy metabolism, and delay senescence-associated chlorophyll degradation in grapes, while blue light appears to be particularly effective at promoting GABA accumulation and modulating specific aspects of sugar metabolism, highlighting spectrum-dependent partitioning of metabolic responses.^62^

A similar emphasis on energy metabolism is evident in the work of Zhou et al., who explored how continuous red LED illumination (655 ± 1 nm, 800 ± 10 lux) affects the postharvest ripening of mature-green banana (Musa spp., cv. “Brazil”) compared to dark storage and ethephon treatment (500 mg/L) at 20 °C ± 0.1 °C and 75% ± 5% relative humidity over eight days. Red light accelerated ripening in a manner comparable to that of ethephon, advancing respiratory and ethylene production peaks from day 6 to day 4, hastening the peel color transition from green to yellow, reducing firmness, and promoting earlier accumulation of soluble solids. Red LED treatment also enhanced chlorophyll degradation and carotenoid biosynthesis, leading to higher carotenoid levels (23.04 μg/g by day 8) than those observed in ethephon-treated fruits (19.54 μg/g), suggesting at least comparable efficacy in color development. Mechanistically, red light markedly increased ATP content and sustained higher ATP levels throughout storage while elevating activities of H⁺-ATPase, Ca²⁺-ATPase, cytochrome c oxidase, and succinate dehydrogenase; organic acid degradation, particularly that of quinic and malic acids, was also accelerated via increased malic enzyme and NADP-malic enzyme activities, providing additional substrates for respiration and ATP production. Zhou et al. therefore proposed that red light promotes banana ripening primarily through the stimulation of energy-generation pathways and respiratory activity rather than solely through ethylene enhancement and suggested that red LED treatment may serve as a non-chemical alternative to ethephon for controlling banana ripening, although broader validation under commercial conditions remains necessary.^60^

In contrast to red light-driven acceleration of ripening in bananas and enhanced phenolic and energy metabolism in grapes, Lu et al. examined how blue LED irradiation (446 nm, 35 μmol m^−2^ s^−1^) combined with low-temperature storage (8 °C) influences postharvest strawberry (*Fragaria *×* ananassa “Benihoppe”*) quality, focusing specifically on antioxidant defense and cell wall metabolism over 10 d. Strawberries treated with blue light at both 22 °C and 8 °C maintained higher firmness, total phenol content, and soluble sugar levels than dark-stored controls, with the strongest effects under blue light + 8 °C, where phenolics and soluble sugars increased by approximately 28.4% and 22.8%, respectively. Blue light reduced respiration and ethylene production, lowered oxidative stress markers such as malondialdehyde, superoxide anion, and hydrogen peroxide, and enhanced enzymatic antioxidant systems (SOD, POD, CAT, PAL, APX, and GR) as well as non-enzymatic antioxidants (ascorbic acid, glutathione, and total phenolics), indicating a reinforced antioxidant network. At the cell wall level, blue light preserved protopectin, cellulose, and hemicellulose contents by suppressing cell wall-degrading enzymes, including polygalacturonase, cellulase, pectin methylesterase, and β-glucosidase, thereby delaying softening and structural degradation; integrated membership function analysis of 25 indicators identified blue light + 8 °C as the most effective treatment (score 0.60) for preserving overall quality. This study shows that, in strawberry, blue light applied during storage primarily slows ripening and senescence by strengthening antioxidant and cell wall integrity systems, complementing the delayed ripening and enhanced anthocyanin retention observed under blue-enriched light in purple peppers by,^59^ while contrasting with the ripening-accelerating effects of red light in bananas and phenolic- and energy-promoting roles of red light in grapes.^58^

Beyond fruit, Zhang and Xie investigated red and violet LED effects on fresh-cut pakchoi (*Brassica rapa* L. *chinensis*) stored at 4 °C, emphasizing the physiological quality, antioxidant defense, and microbial communities, thereby illustrating how LED treatments can simultaneously modulate plant physiology and surface microbiota in minimally processed vegetables. Fresh-cut samples subjected to darkness, violet (405 nm; T1), red (660 nm; T2), or combined red–violet (405 + 660 nm; T3) irradiation (15 μmol m^−2^ s^−1^, 12 h/d, 12 d) showed that T3 best maintained chlorophyll and soluble solids, reduced yellowing, and preserved AsA content, sensory attributes, and overall quality scores compared with controls. Antioxidant enzyme activities (SOD, POD) were significantly increased under the LED treatments, suggesting improved ROS-scavenging capacity, while shelf-life was extended from 8 d in controls to up to 13 d under combined red–violet light. Importantly, LED irradiation, particularly T3, suppressed the proliferation of spoilage-associated Pseudomonas spp., whose relative abundance reached 95.73% in controls but remained much lower in the T3-treated samples, and achieved microbial reductions comparable to those of sodium hypochlorite (~1 log CFU/g) without chemical residues. Although this study focuses on leafy vegetables rather than fleshy fruits, it underscores that LED-mediated preservation may act not only via modulation of plant metabolism and antioxidant systems but also through microbiome shifts, providing a broader context for interpreting fruit-based findings where microflora are rarely characterized in detail.^64^

In addition to direct modulation of ripening and metabolism by LEDs, optical techniques using LED excitation have been explored for non-destructive assessment of fruit ripeness, thereby enabling more precise application of light-based preservation strategies. Lin et al. used fluorescence and reflectance spectroscopy with UV/visible LED excitation (365, 385, 395, 405 nm) to monitor avocado (cv. unspecified) ripening under different storage regimes (room temperature ~25 °C, 9 °C refrigeration, or combined treatments), aiming to develop a low-cost, practical ripeness assessment tool. They quantified chlorophyll-related reflectance ratios (R670/R750, R550/R750) and fluorescence ratios (I740/I685, I550/I685), observing that chlorophyll degradation during ripening increased the reflectance ratios and altered the fluorescence, with F1 (I740/I685) decreasing and F2 (I550/I685) increasing over time; room temperature storage produced the strongest spectral dynamics, whereas refrigerated fruits showed slower changes. The authors also noted considerable intra-fruit spectral variability linked to differential sunlight exposure during growth, which they mitigated by averaging measurements from opposite positions, reducing variability from 190%–350% to 15%–20% and improving ripening prediction accuracy to approximately one day. Among the tested wavelengths, 405 nm excitation produced the most pronounced fluorescence changes, leading to the proposal of a handheld fluorosensor comprising a 405 nm LED, detectors, filters, and a microprocessor and the demonstration of remote fluorescence sensing using a 1 W 412 nm laser and telescope at a 10 m distance; these findings suggest that LED-based fluorescence diagnostics could be integrated into postharvest workflows for non-destructive ripeness monitoring in avocados and potentially other fruits.^56^

Similarly, Panebianco et al. evaluated delayed luminescence (DL) as a rapid, non-destructive optical method for assessing tomato (*cv. Creativo*) maturity and postharvest ripening, focusing on how DL signals, which arise from long-lived excited states, change as fruits progress through four ripening classes (*fully red, fully orange/light red, orange–green*, and *green*) during storage at 20 °C ± 2 °C and 65% relative humidity. While the CIE a*/b* color ratio increased with ripening and served as a reliable index of pigment transition from chlorophyll to lycopene, the DL intensity decreased with ripening, with immature green fruits exhibiting higher photon emission and fully ripe fruits showing lower DL, which was consistent with chlorophyll loss and carotenoid accumulation. Panebianco et al. further noted that DL patterns differed between field-ripened and postharvest-ripened tomatoes even when the external color was similar, indicating that DL can capture differences in internal physiological states not evident from color alone; total soluble solids were also higher in fully ripe class A fruits (approximately 7.1–8.1 °Brix), reinforcing the distinction in internal quality among classes. Taken together,^56^ these optical studies highlight that LED-based or LED-excited techniques can complement direct LED treatments by providing non-destructive tools to classify ripening stages, track chlorophyll and carotenoid dynamics, and discriminate between naturally and artificially ripened fruits, thereby supporting more targeted deployment of light-mediated preservation strategies.^57^

Collectively, these studies indicate that light-mediated control of fruit ripening, softening, and energy metabolism is highly crop- and context-dependent, with red and blue spectra exerting distinct, and sometimes opposing, effects on the ripening rate, pigment dynamics, antioxidant capacity, and cellular energy status across species. While red light often accelerates climacteric ripening or enhances phenolic and energy metabolism (as in bananas and grapes), and blue-enriched light tends to delay ripening while promoting anthocyanin accumulation and strengthening antioxidant and cell wall integrity (as in purple peppers and strawberries), the mechanistic bases remain only partly resolved and are frequently inferred from a limited set of biochemical or transcriptional markers rather than comprehensive multi-omics analyses or direct causal tests. Important limitations include the predominance of controlled-environment experiments with relatively small sample sizes, simplified storage conditions, and fixed, sometimes narrow, ranges of light intensity and spectral composition, which complicates extrapolation to commercial supply chains where light exposure interacts with variable temperature, humidity, handling, and microbiological loads. Moreover, many studies focus on short storage periods and single cultivars and rarely consider trade-offs such as energy costs, potential negative effects on flavor development or consumer acceptance when ripening is strongly delayed, or spectrum-specific shifts in surface microbiota that may alter safety and spoilage patterns. Future work should therefore prioritize systematic, multi-factorial trials that integrate the light spectrum, intensity, photoperiod, and temperature across diverse cultivars and commodity classes; combine physiological, biochemical, and high-throughput molecular analyses to resolve how photoreceptor signaling, energy metabolism, and hormone networks converge under postharvest conditions; and extend to pilot- or full-scale demonstrations that quantify economic feasibility, energy use, and compatibility with existing cold-chain infrastructure and packaging. Such efforts, ideally guided by non-destructive optical tools capable of real-time ripeness and quality assessment, will be essential to translate promising laboratory findings into robust, scalable LED-based strategies for managing ripening, maintaining textural integrity, and optimizing nutritional and sensory quality in commercial postharvest systems.

## LED/UV technologies for preservation, safety, and quality monitoring

Light-emitting diode (LED) and ultraviolet LED (UV-LED) technologies have emerged as multifunctional and non-thermal tools for improving the postharvest safety, quality, and longevity of horticultural commodities. As summarized in Table 6, various LED and UV-LED light treatments applied across different crops and systems demonstrate wavelength-dependent effects on the microbial load, physiological responses, and quality attributes. These technologies serve multiple roles, ranging from microbial disinfection and modulation of plant metabolism to the enhancement of bioactive compounds and maintenance of visual and nutritional quality. Unlike conventional approaches, LEDs allow fine-tuned control of spectral composition, intensity, and exposure duration, facilitating targeted and energy-efficient intervention. However, given the strong dependence of outcomes on wavelength and commodity type, a deeper mechanistic understanding remains crucial for their effective and scalable implementation in postharvest systems.

**Table 6.** Overview of LED and UV-LED treatments in horticultural commodities, summarizing wavelength conditions and their reported effects on microbial load, physiological responses, and quality attributes. These studies demonstrate the multifunctional potential of light-based technologies in postharvest preservation, including disinfection, enhancement of bioactive compounds, and quality retention. Upward (↑) and downward (↓) arrows denote increases and decreases in the respective parameters.

Crop	Light treatment	Reported outcomes	Reference
Pomegranate (*Punica granatum* L.)	Deep UV-LED (255 ± 1 nm)	↓ microbes (bacteria, molds, yeast); ↑ anthocyanins, polyphenols, ascorbic acid, antioxidant capacity	[66]
Tomato	Violet LED (405 nm)	↓ *Botrytis cinerea*, *Rhizopus stolonifer*; ↑ phenolics, vitamin C, antioxidants; ↑ mass loss; ↓ lycopene	[67]
Ethylene sensing system	UV light (365 nm)	↑ detection sensitivity (to 5 ppm)	[68]
Strawberry (*Fragaria* × *ananassa*)	Broad wavelength range from UVA (350 nm) to near-infrared (1000 nm)	Altered morphology and development	[69]
Broccoli heads (*Brassica oleracea* var. *Italica* cv. Legacy) and kale leaves (*Brassica oleracea* L. convar. *Acephala*)	White LED (moderate intensity)	↓ senescence; ↑ chlorophyll retention, color, sugars	[70]
Pepper (*Capsicum annuum* L.)	Red–blue LED (660 nm dominant)	↑ photosynthesis; improved leaf structure	[71]
Chili pepper (*Capsicum annuum* L.)	Red (660 nm), Blue (450 nm), White LEDs	Red/blue: ↑ capsaicinoids; white/red: ↑ amino acids	[72]
Fresh-cut potato (*Solanum tuberosum* L.)	420 nm LED + curcumin	Inactivation of E. coli and S. aureus; maintained quality	[73]
Litchi (*Litchi chinensis* Sonn.)	410–420 nm LED (through packaging)	↓ fungal conidia (Fusarium, Geotrichum)	[74]

In this context, Chua et al. investigated the antifungal efficacy of postharvest 405 nm LED illumination on tomato fruit and simultaneously assessed its influence on physicochemical quality under a meso-scale storage system. In their study, tomatoes inoculated with *Botrytis cinerea* and *Rhizopus stolonifer* were continuously exposed to 405 nm light at an irradiance of 87 W/m² for 12 d under controlled storage conditions of 15 °C and 80% relative humidity. The treatment produced a pronounced antifungal effect, reducing fungal populations by 1.9 log CFU/g for *B. cinerea* and 3.2 log CFU/g for *R. stolonifer*, equivalent to approximately 98.7% and 99.9% reductions, respectively. The authors attributed this effect to photodynamic inactivation, whereby 405 nm light excites endogenous porphyrins in fungal cells and induces reactive oxygen species formation, ultimately damaging cellular structures. Importantly, the treatment was not only antimicrobial but also altered fruit quality responses. LED-exposed tomatoes showed increased total phenolic content, antioxidant activity, and vitamin C levels during later storage, suggesting stimulation or preservation of antioxidant metabolism. However, these benefits were accompanied by greater weight loss and reduced lycopene accumulation after prolonged storage, even though color, texture, glucose, and fructose were largely unaffected. Thus, this study indicates that 405 nm LED light can be an effective non-chemical strategy for suppressing fungal spoilage in tomatoes, but it also reveals the typical postharvest trade-off in which improved microbial control and antioxidant status may come at the expense of certain compositional or water-retention attributes.^67^

X. Yu et al. evaluated whether LED-based antifungal treatments remain effective in packaged systems, which is highly relevant because many postharvest commodities are marketed in enclosed plastic films. Their work focused on litchi fruit and examined the penetration and antimicrobial efficacy of violet (410–420 nm), blue (460–470 nm), and green (520–530 nm) LEDs through commercial packaging films, including polyethylene (PE) of different thicknesses, PVC, PET, PVDC, and PA, under refrigerated storage at 4 °C. Both in vitro and in vivo assays were conducted using *Fusarium sp.* and *Geotrichum candidum* isolated from infected litchi; fungal suspensions were applied onto agar media and fruit surfaces, light transmission through films was quantified, and antifungal performance was evaluated by colony counting and first-order inactivation kinetics. Although all the films transmitted a high proportion of incident light (approximately 88%–95%), violet light consistently showed the strongest antifungal effect, with inhibition rates of about 52%–83%, compared with 11%–23% for blue and 4%–15% for green. Packaging reduced the efficacy relative to direct illumination by roughly 8%–35%, but thin PE, PVC, and PET films caused the smallest losses, allowing complete inhibition of *Fusarium sp.* after 12 h of violet LED exposure, whereas a thicker PE and PA required more than 16 h; G. candidum was more sensitive and was fully inhibited within 6–8 h, depending on the film. In inoculated litchi, direct violet LED reduced Fusarium sp. and G. candidum by up to 2.24 and 2.58 log CFU/g after 72 h, and packaged fruit also showed substantial reductions, particularly under PET and PVC. From a quality standpoint, direct LED exposure increased weight loss and color change, whereas packaging, especially thin PE and PVC, maintained weight loss below 10% and limited Δ*E* to minor, barely perceptible changes. The authors emphasized that antimicrobial efficacy depended more on the spectral match between violet light and microbial endogenous photosensitizers, such as porphyrins absorbing in the 400–430 nm range, than on the transmission percentage alone and noted that condensation or fogging on films could further diminish the effective dose. Overall, this study extends the findings of Chua et al. by demonstrating that violet LEDs can still achieve meaningful fungal control in packaged fruit, but it also underscores that exposure time, film selection, and moisture-related optical interference are critical parameters for practical implementation.^74^

Whereas the first two studies focused mainly on fungal suppression in whole fruits, Aihaiti et al. demonstrated that UV-LEDs can also be used in minimally processed products where safety and nutritional retention are equally important. They investigated the effect of UV-LED treatment on ready-to-eat pomegranate arils using a chamber equipped with 200 UV-LED bulbs operating at 255 ± 1 nm with an irradiation intensity of 2.1 ± 0.1 mW/cm² for 10 min, followed by storage at 4 °C for 5 d. Compared with untreated controls, UV-LED-treated arils showed clear improvements in both microbiological and biochemical parameters. Aerobic plate counts and mold/yeast populations were reduced by 1.05 and 0.55 log CFU/g, respectively, and remained below the safety threshold during storage. At the same time, anthocyanin, polyphenol, ascorbic acid, and antioxidant capacity increased and peaked on day 3, whereas those of the control samples showed declining trends. Relative to controls, the anthocyanin and polyphenol contents increased by 0.06 and 0.15 mg/g, respectively, on day 3. To explain these responses, the authors performed untargeted LC–MS metabolomics combined with PCA, OPLS-DA, and KEGG pathway enrichment, which revealed significant shifts in the flavonoid and anthocyanin biosynthesis pathways. Seven anthocyanin-related metabolites were upregulated, among which peonidin-3-O-rutinoside chloride increased the most dramatically, by 46.46-fold. This mechanistic layer makes the study particularly valuable because it moves beyond descriptive quality outcomes and shows that UV-LED treatment may actively reprogram secondary metabolism while simultaneously reducing microbial contamination. In that sense, Aihaiti et al. complemented antifungal studies on tomato and litchi by demonstrating that short-wave UV treatment can achieve a dual benefit in fresh-cut products, namely, decontamination together with stimulation of health-promoting metabolites.^66^

A different dimension of LED application was explored by Pintos et al., who examined how continuous white LED illumination at different intensities affects postharvest senescence and quality in broccoli and kale during refrigerated storage. Freshly harvested broccoli and green and red kale were stored at 5 °C and 90% ± 1% relative humidity either in darkness or under continuous white LED light at low, medium, and high intensities of 10, 30, and 80 μmol m^−2^ s^−1^, corresponding to approximately 740, 2300, and 6000 lux, respectively, generated by white LED panels with a color temperature of 4400 K. Broccoli were stored for 17 d, and kale for 11 d. In contrast to studies emphasizing microbial inactivation, this work focused on senescence-related visual and biochemical quality traits. The results showed that light intensity was a decisive factor, with a medium intensity of 30  μmol/m² s^−1^ producing the most favorable responses. Under this treatment, yellowing was delayed, the total color change was reduced, hue values were better maintained, and chlorophyll retention was substantially improved. Broccoli and green kale retained about 30%–35% more chlorophyll than the dark-stored controls, while red kale maintained nearly 2.5 times higher chlorophyll content. In addition, this intermediate light level reduced sugar loss and better preserved carotenoid and antioxidant contents. In contrast, both the low- and high-intensity treatments were less beneficial and led to greater weight loss, with the high-intensity treatment potentially imposing oxidative stress and the low-intensity regime likely being insufficient to sustain the desired metabolic status. Principal component analysis further confirmed that the mid-intensity treatment maintained the samples closest to their initial visual and biochemical condition. The study thus highlights an important principle that is also implicit in earlier tomato and litchi work: LED efficacy is not simply a matter of exposure versus no exposure but of optimizing intensity to balance beneficial physiological signaling against stress induction.^70^

The importance of spectral optimization is further supported by the work of Liu et al., who evaluated postharvest LED irradiation in chili pepper fruit with emphasis on firmness, bioactive compounds, and amino acid composition. Mature green fruits from three cultivars, “Hangjiao-2”, “Xinxiang-2”, and “P1622”, were exposed for 48 h at 24 °C to continuous white light, red light at 660 nm, or blue light at 450 nm, each at 50 μmol m^−1^ s^−1^, with dark storage as the control. Their results showed that red and blue light were particularly effective in enhancing capsaicinoid accumulation, with capsaicin content increasing by up to 88% and dihydrocapsaicin also increasing substantially, especially in “P1622”. At the same time, white and red light improved the proportion of essential and aromatic amino acids, whereas blue light increased monosodium glutamate-like amino acids in certain cultivars, indicating that spectral quality can modulate not only phytochemical content but also flavor-associated amino acid profiles. Additional effects were observed in chlorophyll, carotenoid, vitamin C, and soluble protein contents, but these responses varied with genotype. The physical quality also responded differently to the spectral treatments, since white light generally reduced firmness, whereas red and blue light improved firmness in some cultivars. By identifying 17 amino acids, including essential and flavor-related forms, this study provides a broader nutritional interpretation of LED effects than studies focused only on pigments or antioxidants. It also reinforces a recurrent theme across the literature: the outcome of light treatment depends strongly on genotype and target trait, so successful postharvest application requires matching wavelength to commodity-specific metabolic priorities rather than expecting a single spectrum to optimize all quality parameters simultaneously.^72^

A more explicitly antimicrobial and mechanistically integrated strategy was presented by J. Yu et al., who evaluated curcumin-mediated photodynamic treatment (PDT) for fresh-cut potato slices. In this system, 420 nm LED light was used not merely as a direct treatment but also as an activator of edible curcumin, which functioned as a photosensitizer to generate reactive oxygen species for microbial inactivation. Potato slices prepared from uniform tubers were treated with four combinations: light plus curcumin (PDT), light alone, curcumin alone, and the untreated control. The selected conditions were 30 μmol L^−1^ curcumin and 20 min exposure to a 420 nm LED system delivering 0.7 kJ cm^−2^. *Escherichia coli* and *Staphylococcus aureus* were inoculated into the samples, and changes were monitored over 8 d at 4 °C, including microbial counts, color, texture, and total soluble solids, weight change, antioxidant capacity, total phenolics, total flavonoids, and the activities of PPO, POD, and PAL. The PDT treatment produced clear bactericidal effects, reducing *E. coli* by 2.43 log CFU mL^−1^ and *S. aureus* by 3.18 log CFU mL^−1^, with the greater sensitivity of *S. aureus* attributed to the higher permeability of Gram-positive cell walls to curcumin. Importantly, this microbial reduction was achieved without major deterioration in visual quality, texture, or soluble solids. Moreover, the PDT-treated samples showed reduced weight gain, increased total antioxidant capacity, and slower losses of total phenolics and flavonoids. Mechanistically, PPO and POD activities were immediately reduced by about 59.7% and 47.8%, while PAL activity increased, suggesting that the treatment simultaneously suppressed oxidative degradation and stimulated phenolic biosynthesis. This study is particularly relevant because it bridges microbial decontamination and quality maintenance through an enzymatic explanation, thereby offering a more integrated framework than simple microbial kill data alone. In relation to the previous studies, it also shown that visible LEDs can become substantially more effective when combined with compatible photosensitizers, opening a route for stronger antimicrobial outcomes under relatively mild treatment conditions.^73^

Although not strictly a postharvest preservation study, the work of Sobczak et al. is useful for understanding how LED spectra influence structural and physiological traits that may subsequently affect postharvest performance. They compared supplementary lighting from high-pressure sodium lamps and red–blue LEDs in pepper seedlings of the cultivars “Aifos” and “Palermo” under greenhouse conditions. Seedlings were grown under similar photosynthetically active radiation levels of about 170 μmol m^−2^ s^−1^ with a 16 h photoperiod, while the LED treatment consisted of 87.5% red light at 630–660 nm and 12.5% blue light at 440–460 nm. LED supplementation improved growth in a cultivar-dependent manner, particularly increasing seedling height and leaf number in “Aifos”. It also increased the chlorophyll content and photosynthetic performance index, with the PI rising by about 45% in “Aifos” and 30% in ‘”alermo” compared with HPS-grown plants. Anatomical analyses revealed thicker leaves, larger palisade cells, thicker spongy mesophyll, and greater stomatal density under LEDs, while ultrastructural observations showed larger and more abundant chloroplasts, thicker grana, and higher starch accumulation. Confocal fluorescence imaging further showed stronger chlorophyll and carotenoid signals and improved pigment colocalization. These findings suggest that LED spectra can modify leaf architecture and the photosynthetic machinery in ways that enhance light capture and assimilation efficiency. Although this study concerns preharvest growth rather than direct postharvest treatment, it contributes mechanistic insight relevant to other studies by illustrating how spectral composition shapes tissue structure, pigment organization, and energy metabolism, which may in turn influence later storage behavior and responsiveness to postharvest lighting.^71^

This broader systems-level perspective is further expanded by Lu et al., who investigated full-spectrum LED lighting in strawberries under controlled-environment agriculture and combined classical correlation analysis with deep-learning-based time-series prediction. A total of 500 strawberry plants were grown under 72 dynamic light-quality combinations spanning 350–1000 nm, including UVA, violet, indigo, blue, green, yellow, orange, red, and near-infrared wavelengths, with overall light intensities maintained at 250–300 μmol m^−2^ s^−1^ for approximately 200 d. Nine traits were monitored, including plant height, leaf number, leaf area, stolon development, flowering time, fruit number, fruit ripening time, total soluble solids, and fruit skin hardness. Spearman's rank correlation analysis showed a clear division between the growth-promoting and quality-promoting spectral zones: red and near-infrared wavelengths strongly supported vegetative growth, leaf expansion, and stolon development, whereas shorter wavelengths, such as UVA and blue generally suppressed vegetative growth but enhanced fruit quality indicators, including total soluble solids and skin hardness. The study then integrated CEEDMAN preprocessing with an Informer neural network model, which outperformed Transformer-based methods in predicting trait responses across changing spectral conditions. The authors suggested that short wavelengths may regulate antioxidant and secondary metabolite responses through photoreceptor-mediated pathways, while red and near-infrared wavelengths act more strongly through phytochrome-mediated growth regulation. Although centered on cultivation rather than postharvest storage, this work is highly relevant because it supports the idea that optimal LED application should be treated as a multidimensional design problem rather than a single-factor treatment and that predictive modeling may become essential for tailoring spectral strategies to desired outcomes in quality preservation systems.^69^

Finally, Jaisutti et al. moved beyond direct preservation and addressed quality monitoring by developing a UV-activated room-temperature ethylene sensor for fruit-ripeness detection and supply-chain management. Their device consisted of silver nanoparticle-decorated ZnO nanoflowers fabricated on patterned ITO interdigital electrodes operated under continuous 365 nm UV LED illumination positioned 1 cm from the sensing surface. The UV intensity was optimized, and 5 mW/cm² yielded the best performance. Under these conditions, the Ag10/ZnO sensor achieved a response of 52.83% to 40 ppm ethylene, about four times higher than that of the pristine ZnO sensors, and displayed a linear response from 10 to 70 ppm with an *R*² of 0.9892. The device also showed a low detection limit of 5 ppm, response and recovery times of approximately 9.8 and 7.5 min, repeatability with less than 1.3% variation, and stability over 90 d. The enhanced sensitivity was attributed to the combined catalytic role and localized surface plasmon resonance effect of silver nanoparticles under UV excitation, which improved oxygen adsorption and charge transfer. Practical relevance was demonstrated using banana ripening, in which the sensor response rose progressively and peaked around day 7, confirming its ability to monitor ethylene evolution and fruit maturity in real time. In relation to the preceding preservation studies, this work broadens the functional role of LED and UV-LED technologies from active intervention to intelligent monitoring, suggesting that future postharvest systems may integrate light-based disinfection, metabolic regulation, and sensing into unified smart storage platforms.^68^

Collectively, these studies confirm that LED and UV-LED technologies provide a versatile, non-thermal platform for postharvest preservation, enabling wavelength-specific control over microbial safety, senescence, and the accumulation of health-promoting metabolites, and, in the case of UV-activated ethylene sensors, real-time quality monitoring. At the same time, several limitations constrain immediate large-scale adoption: most experiments are conducted under highly controlled laboratory or meso-scale conditions, often with continuous illumination and single commodities, so their transferability to heterogeneous, industrial supply chains with fluctuating temperature, humidity, and loading patterns remains uncertain; treatment windows are typically narrow, with efficacy strongly dependent on precisely tuned wavelength, intensity, exposure duration, and commodity genotype, and trade-offs such as increased weight loss, altered pigment development, or firmness changes become evident when conditions deviate from this optimum. Packaging adds another layer of complexity, as shown by violet LED treatments in litchi, where film type, thickness, and surface condensation can substantially modify the effective dose and antifungal outcome, highlighting that optical compatibility and moisture control must be co-optimized alongside spectral design. Future research should therefore move beyond descriptive “one-factor-at-a-time” trials and adopt more integrative, mechanistic frameworks that explicitly couple photobiology, microbiology, and technology engineering, for example, combining LED spectra with photosensitizers or metabolomic readouts, using dynamic or pulsed light regimes instead of continuous exposure, and leveraging predictive models to tailor spectral–dose combinations to specific commodities and storage stages. In parallel, there is a need for pilot- and commercial-scale demonstrations that address practical constraints such as energy use, cost–benefit trade-offs versus conventional treatments, regulatory acceptance of UV and photodynamic interventions, and the robustness of LED-based systems in real packaging and logistics environments. By systematically addressing these limitations and aligning mechanistic understanding with engineering optimization, LED/UV technologies can evolve from promising experimental tools into reliable, scalable components of smart postharvest preservation and monitoring systems.

## Challenges and future research directions

**Figure 8.**
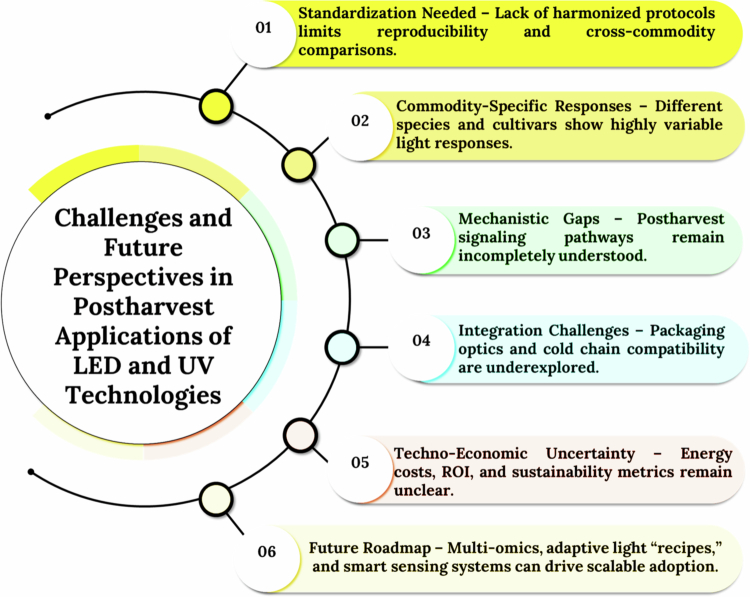
Key challenges and future research directions in the application of LED and UV technologies for postharvest preservation. Major limitations include a lack of standardized protocols, strong commodity- and cultivar-specific responses, incomplete understanding of underlying molecular mechanisms, and limited integration with commercial storage systems. Additional uncertainties related to techno-economic feasibility, energy costs, and scalability remain. Future research should focus on multi-omics approaches, dynamic spectral “light recipes,” and integration with smart sensing systems to enable reproducible and scalable applications. Image created with www.biorender.com.

Despite substantial advances in the application of LED and UV technologies across preharvest and postharvest systems, several key challenges continue to limit mechanistic clarity, reproducibility, and large-scale implementation, as shown in Figure 8. One of the most prominent limitations is the pronounced variability in responses across species, cultivars, and developmental stages. Evidence across diverse commodities, including tomato, strawberry, pepper, grape, citrus, leafy vegetables, and berries, consistently demonstrates that identical light spectra and exposure regimes can produce markedly different, and sometimes opposing, physiological and biochemical outcomes. For instance, blue or blue-enriched light frequently enhances phenolic and anthocyanin accumulation, yet its effects on ripening, sugar metabolism, firmness, and carotenoid content remain highly context-dependent and contradictory across cultivars and developmental stages.^23^ ^,^ ^29^ ^,^ ^59^ Similarly, red and far-red wavelengths have been shown to promote fruit sink strength, yield, or ripening in certain systems while simultaneously reducing pigment accumulation or accelerating senescence in others.^19^ ^,^ ^20^ ^,^ ^60^ Critically,^18^ demonstrated in a three-year greenhouse trial that LED lighting increased β-carotene in Chocomate tomato by 34.3% but reduced it in Bolzano by 18.5%, underscoring that cultivar selection, rather than spectral quality alone, is often the primary determinant of outcome. Likewise,^23^ showed that green-mature cherry tomatoes responded far more strongly to both red and blue wavelengths than turning-stage fruit, confirming that the developmental stage at the time of light application profoundly modulates responsiveness. These inconsistencies collectively indicate that current knowledge remains largely descriptive and lacks predictive frameworks capable of accounting for genotype-specific light responsiveness, thereby limiting the transferability of findings across horticultural systems. Rather than treating these discrepancies as contradictions, future research should interpret them as evidence that universal spectral prescriptions are premature and that cultivar- and stage-resolved investigations using standardized parameters are essential for identifying reliable biomarkers of light sensitivity, such as photoreceptor expression profiles or phytochrome photostationary state, that can inform genotype-tailored spectral prescriptions.

A second major constraint lies in the fragmented mechanistic understanding of light-mediated regulation, particularly in postharvest tissues. Although many studies have reported associations between spectral treatments and changes in antioxidants, pigments, sugars, hormones, and redox balance, the underlying signaling networks are not fully resolved. Key regulatory components, including photoreceptors and transcription factors such as HY5, HYH, MYBs, PIFs, and ERFs, have been identified as central nodes linking light perception to metabolic responses, yet their integration with hormone signaling, sugar sensing, ion homeostasis, and energy metabolism remains incomplete and is often inferred from single-species studies.^30^ ^,^ ^32^ ^,^ ^33^ This limitation is especially evident in harvested organs, where metabolic regulation may differ fundamentally from actively growing tissues and where photosynthesis-independent signaling predominates. A striking illustration of this gap is provided by^26^ who showed that elevated PSY, LCY, OHASE1, and CCD4 transcripts under blue–white LED in pakchoi sprouts translated into net carotenoid accumulation only when non-blue wavelengths were simultaneously present, demonstrating that transcript abundance alone cannot predict metabolic flux and that the spectral context is biochemically decisive. Similarly,^25^ implicated ERF021 as a central regulatory hub linking light signaling to metabolic shifts in hot pepper, yet the expected downstream upregulation of amino acids and carbohydrates did not materialize, leaving the proposed causal pathway unresolved. Furthermore, reports of improved quality coinciding with reduced ATP levels or altered energy charge raise unresolved questions regarding the role of metabolic downregulation in delaying senescence.^52^ These examples collectively demonstrate that mechanistic claims in the current literature are frequently inferential, based on correlations between gene expression patterns established in model species and biochemical outcomes in less-characterized crops, without direct functional validation. Addressing these gaps will require coordinated, multi-omics approaches, integrating transcriptomics, metabolomics, proteomics, and ionomics, applied systematically across developmental stages and storage conditions in postharvest-specific contexts, rather than isolated mechanistic observations extrapolated from pre-harvest or model-plant studies.

Methodological heterogeneity represents another critical barrier to progress. Current studies vary widely in spectral composition, wavelength bandwidth, irradiance, photoperiod, exposure duration, timing of application, and storage conditions. Even within broadly defined spectral categories such as “blue” or “red” light, substantial differences in peak wavelengths and intensities hinder direct comparison across studies.^36^ ^,^ ^44^ ^,^ ^45^ This variability is further compounded by interacting environmental factors, including temperature, humidity, atmospheric composition, packaging materials, and microbial load, all of which can significantly influence treatment outcomes. For example, while^58^ showed that blue LED at 446 nm combined with 8 °C storage strongly preserved strawberry firmness and antioxidant status, other studies employing different peak wavelengths or higher irradiances on different cultivars reported variable effects on firmness and increased weight loss, differences that cannot be meaningfully interpreted without uniform reporting of all treatment parameters. Notably, light–temperature interactions can either enhance or offset physiological responses, yet these factors are rarely evaluated in integrated experimental designs.^38^ ^,^ ^73^ Most studies also rely on small sample sizes, single cultivars, and short experimental windows, restricting generalizability and making it difficult to disentangle genuine biological variation from context-dependent artefacts. The absence of standardized reporting frameworks for light treatments, specifying at minimum the full spectral output of the light source, photon flux density at the product surface, total energy dose, temperature and humidity, cultivar name and ripening stage, and packaging type, therefore, remains a major obstacle to reproducibility, meta-analysis, and evidence-based protocol development. Establishing such a framework through a community-led initiative would dramatically improve cross-study comparability and accelerate the identification of robust, crop-specific spectral regimes.

A recurrent but insufficiently analyzed theme is the existence of significant trade-offs between different quality attributes under identical light treatments, which challenges the assumption that optimizing one parameter automatically benefits overall postharvest quality.^20^ showed that far-red supplementation of red–blue LEDs interlighting increased total sweet pepper fruit yield by up to 19%, yet simultaneously reduced total carotenoids by 18%–24%, imposing a direct trade-off between productivity and nutritional density that growers must explicitly weigh. Similarly,^67^ demonstrated that 405 nm violet LED illumination effectively reduced fungal populations of *Botrytis cinerea* and *Rhizopus stolonifer* in tomato by 98.7% and 99.9%, respectively, and enhanced phenolic content and antioxidant activity, yet also caused greater weight loss and reduced lycopene accumulation during prolonged storage. In the context of UV-LED applications, the narrow therapeutic window between effective pathogen inactivation and potential tissue damage represents a further operational trade-off that constrains dose optimization in commercial settings.^42^ ^,^ ^43^ These findings collectively indicate that postharvest light optimization cannot be framed as a single-parameter maximization problem but must instead be approached as a multi-objective challenge in which the simultaneous effects on appearance, texture, nutritional composition, antimicrobial safety, energy cost, and commercial viability are evaluated together. Future studies should therefore explicitly quantify trade-offs rather than reporting only beneficial outcomes and should adopt composite quality indices, such as the integrated membership function approach used by,^43^ which allows holistic comparison across multiple traits simultaneously.

From a translational perspective, the practical implementation of LED and UV technologies in commercial postharvest systems remains substantially underexplored. While many studies have demonstrated promising results under controlled laboratory conditions, few have assessed long-term performance under commercial storage, transport, and retail environments, where the temperature fluctuates, the humidity varies, the produce is mixed, and the light exposure is intermittent.^46^ Packaging adds a further layer of complexity: as demonstrated by^74^ in litchi, the type, thickness, and surface condensation of packaging films can substantially modify the effective photon dose delivered to the product, reducing antifungal efficacy by 8%–35% depending on the film type. The engineering challenges related to uniform light delivery across heterogeneous, bulk-stored produce also remain unresolved, as light penetration to inner layers of stacked bins or crates is severely limited and may create inconsistent quality gradients across the product lot. Critical factors such as energy consumption, system durability, cost–benefit balance, and compatibility with existing cold-chain infrastructure are rarely evaluated alongside biological outcomes.^42^ Although UV-LED systems, particularly in the UVC range, exhibit strong antimicrobial efficacy, achieving precise and uniform dose delivery across heterogeneous produce surfaces and high-throughput processing lines remains a significant engineering challenge.^42^ ^,^ ^43^ Regulatory considerations also warrant attention, as UVC systems and photodynamic treatments employing exogenous photosensitizers such as curcumin^73^ may require specific safety assessments and regulatory clearance before deployment in food-handling environments, and future research should therefore document safety profiles alongside efficacy data to support the development of evidence-based operational guidance.

Future research should therefore move beyond descriptive studies toward integrative and application-oriented frameworks that directly address these interconnected challenges. First, comparative, cultivar-resolved investigations using standardized spectral and exposure parameters are essential to distinguish conserved responses from genotype-specific effects and to identify reliable biomarkers of light sensitivity. Second, comprehensive multi-omics approaches integrating transcriptomic, metabolomic, proteomic, ionomic, and redox analyses are needed to establish causal links between light perception and downstream metabolic regulation across both the preharvest and postharvest stages. Third, dynamic and multi-spectral lighting strategies should be prioritized over static, single-wavelength treatments, given evidence that stage-specific light exposure disproportionately influences quality outcomes and that the temporal structuring of light delivery, such as the pulsed blue light regime demonstrated by^27^ can improve quality with lower total photon input than continuous illumination.^24^ Fourth, the integration of LED and UV-LED systems with smart sensing platforms, including UV-activated ethylene sensors^68^ and LED-excited fluorescence tools for non-destructive ripeness monitoring,^56^ should be systematically explored to enable adaptive, feedback-driven light interventions responsive to actual commodity status rather than predetermined treatment schedules. Finally, future studies must extend to real-world conditions, incorporating supply chain variables such as temperature fluctuations, packaging constraints, microbial loads, and storage duration, while also including comprehensive techno-economic and life-cycle assessments to evaluate scalability, energy efficiency, and economic viability across diverse postharvest scenarios. Progress along these dimensions will be essential to move the field beyond empirical, commodity-specific observations toward robust, predictive, and commercially deployable light-based preservation strategies capable of contributing meaningfully to reducing global postharvest food losses.

## Translational and industrial implementation considerations

Although LED- and UV-based technologies have demonstrated promising effects on shelf-life extension, microbial control, and quality preservation under controlled experimental conditions, their transition into routine commercial postharvest practice remains relatively limited. Most available evidence has been generated under laboratory or pilot-scale environments using individual commodities and relatively stable treatment conditions, which may not adequately reflect the operational complexity of commercial supply chains.^46^ Large-scale storage and distribution systems involve variable temperature conditions, heterogeneous product loads, mixed cultivars, and diverse packaging formats, all of which can influence treatment performance and consistency.

From an engineering perspective, effective implementation requires reliable light delivery across large product volumes. In commercial handling systems, fruits and vegetables are frequently stored in stacked crates, bins, or packaged environments that can create uneven light exposure between the outer and inner product layers. Packaging characteristics, including film composition, thickness, and surface moisture accumulation, may alter photon transmission and reduce treatment efficacy, as demonstrated in packaged litchi systems.^74^ Similar challenges may arise during high-throughput processing operations, where variations in product geometry and surface orientation can produce non-uniform treatment conditions. In addition, commercial processing facilities often operate under high-throughput conditions involving continuous conveyor-based handling systems. Maintaining uniform light dose delivery at industrial processing speeds may require optimized lamp positioning, automated exposure control systems, or multiple irradiation units, potentially increasing equipment complexity. For UV-based systems, particularly within the UVC range, maintaining an effective dose is especially important because antimicrobial efficacy and product quality responses occur within relatively narrow exposure ranges.^42^ ^,^ ^43^ Consequently, practical implementation depends not only on selecting suitable wavelengths but also ensuring reproducible exposure conditions within commercial equipment designs.

Integration with existing postharvest infrastructure also represents an important consideration. Light-based treatments are unlikely to function as completely independent preservation systems and may need to operate in conjunction with established approaches such as refrigeration, controlled-atmosphere storage, and packaging systems. Previous studies have shown that light-mediated responses can vary according to storage temperature and environmental conditions.^37^ ^,^ ^38^ ^,^ ^44^ ^,^ ^45^ These observations suggest that preservation outcomes are influenced by broader storage conditions and may require system-level optimization for practical use. Current implementation may therefore be more practical in relatively controlled environments such as refrigerated storage chambers, packing facilities, and enclosed transportation systems, where light exposure conditions can be more consistently regulated than in open handling systems.

Economic considerations may also influence industrial adoption. Although LED systems generally provide advantages including spectral flexibility, lower heat generation, and improved energy efficiency compared with some conventional lighting technologies,^9^ ^,^ ^10^ commercial implementation may involve additional costs associated with equipment installation, maintenance, system integration, and operational energy requirements. Moreover, some preservation approaches rely on continuous or prolonged illumination periods, which may increase the cumulative energy demand during long-term storage. At present, quantitative cost–benefit assessments relative to conventional approaches remain limited in the literature.^46^ Future implementation studies should therefore incorporate economic metrics such as return on investment, operating costs per unit product, energy consumption, and reductions in postharvest losses to determine practical commercial feasibility. In addition, the economic value of implementation may differ among commodity classes because technologies providing moderate improvements in shelf-life or quality may be more practical for higher-value horticultural products than for commodities with narrower economic margins.

Regulatory and operational considerations may further affect translation into food-processing environments. For visible-light interventions, implementation barriers may be comparatively limited because these systems generally function as physical treatments without introducing chemical residues.^75^ However, UV-based systems, particularly those involving photodynamic approaches using exogenous photosensitizers, may require additional evaluation regarding worker exposure, equipment safety, and food-contact applications before broader deployment.^73^ Regulatory requirements may vary among regions and food sectors because implementation may need to comply with food safety, worker-exposure, and equipment standards established by regional authorities and regulatory agencies. Requirements related to UV exposure limits, food-contact materials, and operational safety may therefore influence commercial deployment. Consequently, broader implementation will likely require evidence that simultaneously addresses preservation performance, operational feasibility, and safety considerations. Overall, current evidence suggests that LED and UV technologies may be more realistically considered complementary components within integrated postharvest systems rather than standalone preservation approaches. Their practical utility will likely depend on compatibility with existing cold-chain infrastructure and on their ability to maintain reproducible performance under realistic storage and transportation conditions.

## Conclusion

Light-emitting diode (LED)- and ultraviolet (UV)-based technologies have emerged as versatile tools for regulating fruit and vegetable physiology across both the preharvest and postharvest stages. The evidence synthesized in this review demonstrates that light quality exerts wavelength-dependent effects on growth, ripening, antioxidant metabolism, microbial control, and the accumulation of bioactive compounds. Across different horticultural commodities, blue- and UV-enriched spectra frequently promote secondary metabolite biosynthesis and antioxidant responses, whereas red and far-red wavelengths more strongly influenced carbon allocation, sink strength, and ripening-related processes. However, these responses are not universally conserved and are strongly influenced by species, cultivar, developmental stage, irradiance, exposure duration, and environmental conditions.

A major insight emerging from this review is that light-mediated regulation cannot be adequately explained by single pathways or individual quality traits alone. Instead, physiological, biochemical, and molecular responses operate through interconnected photoreceptor signaling networks and metabolic processes that collectively determine storage performance and quality outcomes. At the same time, the literature reveals several critical gaps. Considerable variability in experimental design, inconsistent reporting of light parameters, limited mechanistic validation, and reliance on short-term laboratory studies hinder direct comparisons among studies and reduce reproducibility. In addition, most investigations have been performed under controlled conditions, while assessments of scalability, cost-effectiveness, energy requirements, and compatibility with commercial storage systems remain limited.

Future research should prioritize the development of standardized experimental frameworks that include consistent reporting of spectral composition, irradiance, photoperiod, and treatment duration. Longitudinal multi-cultivar studies integrating physiological analyses with transcriptomics, metabolomics, and other systems-level approaches are needed to establish causal mechanisms underlying wavelength-specific responses. Greater emphasis should also be placed on evaluating dynamic and stage-specific lighting strategies rather than static spectral treatments, as well as validating findings under realistic supply-chain and commercial storage conditions. Advancing predictive models that integrate biological responses with technological and economic considerations will be essential for translating laboratory observations into scalable preservation systems. Such efforts may ultimately support the development of crop-specific, energy-efficient, and sustainable light-based strategies for improving postharvest quality and reducing food losses.

## Data Availability

Data sharing is not applicable. No new data generated.

## Abbreviations

ADP: adenosine diphosphate
AMP: adenosine monophosphate
ANS: anthocyanidin synthase
APX: ascorbate peroxidase
AsAGSH cycle/pathway: ascorbate–glutathione cycle
ATP: adenosine triphosphate
bHLH: basic helix–loop–helix transcription factor
CAT: catalase
CHS: chalcone synthase
CmACO1: 1-aminocyclopropane-1-carboxylate oxidase 1 (*Cucumis melo*)
CmPIF8: phytochrome-interacting factor 8 (*Cucumis melo*)
DFR: dihydroflavonol 4-reductase
DHAR: dehydroascorbate reductase
FR: far-red light
GABA: γ-aminobutyric acid
GR: glutathione reductase
HPSL: high-pressure sodium lamp
HY5: elongated hypocotyl 5
LED/LEDs: light-emitting diode(s)
MDHAR: monodehydroascorbate reductase
MdHY5: HY5 transcription factor (*Malus domestica*)
MdHYH: HY5 homolog (*Malus domestica*)
MdPAL: phenylalanine ammonia-lyase (*Malus domestica*)
MdPOD: peroxidase (*Malus domestica*)
MdPPO: polyphenol oxidase (*Malus domestica*)
MeJA: methyl jasmonate
MeSA: methyl salicylic acid
MYB: MYB transcription factor (myeloblastosis family protein)
nm: nanometer
PAL: phenylalanine ammonia-lyase
PPFD: photosynthetic photon flux density
RB/R: B: red: blue light ratio
RBFR: red–blue + far-red light
ROS: reactive oxygen species
SOD: superoxide dismutase
UFGT: UDP-glucose: flavonoid 3-O-glucosyltransferase
UV: ultraviolet
UV-A/UVA: ultraviolet A radiation
UV-B: ultraviolet B radiation
UV-C/UVC: ultraviolet C radiation
UV-LED: ultraviolet light-emitting diode
VvHY5: HY5 transcription factor (*Vitis vinifera*)
VvMYB: MYB transcription factor (*Vitis vinifera*)
VvPIF4: phytochrome-interacting factor 4 (*Vitis vinifera*)
ZnO: zinc oxide
ppm: parts per million
